# RUBIC identifies driver genes by detecting recurrent DNA copy number breaks

**DOI:** 10.1038/ncomms12159

**Published:** 2016-07-11

**Authors:** Ewald van Dyk, Marlous Hoogstraat, Jelle ten Hoeve, Marcel J. T. Reinders, Lodewyk F. A. Wessels

**Affiliations:** 1 Department of Molecular Carcinogenesis, The Netherlands Cancer Institute, Plesmanlaan 121, 1066CX Amsterdam, The Netherlands; 2 Department of EEMCS, Delft University of Technology, Mekelweg 4, 2628CD Delft, The Netherlands

## Abstract

The frequent recurrence of copy number aberrations across tumour samples is a reliable hallmark of certain cancer driver genes. However, state-of-the-art algorithms for detecting recurrent aberrations fail to detect several known drivers. In this study, we propose RUBIC, an approach that detects recurrent copy number breaks, rather than recurrently amplified or deleted regions. This change of perspective allows for a simplified approach as recursive peak splitting procedures and repeated re-estimation of the background model are avoided. Furthermore, we control the false discovery rate on the level of called regions, rather than at the probe level, as in competing algorithms. We benchmark RUBIC against GISTIC2 (a state-of-the-art approach) and RAIG (a recently proposed approach) on simulated copy number data and on three SNP6 and NGS copy number data sets from TCGA. We show that RUBIC calls more focal recurrent regions and identifies a much larger fraction of known cancer genes.

## Abstract (web summary)

Analysis of cancer genome sequencing data has been used to predict genes associated with the pathogenesis of cancer. Here, the authors propose a new algorithm entitled RUBIC that predicts breaks in DNA as opposed to previously published methods that predict amplifications and deletions of DNA.

## Graphical abstract

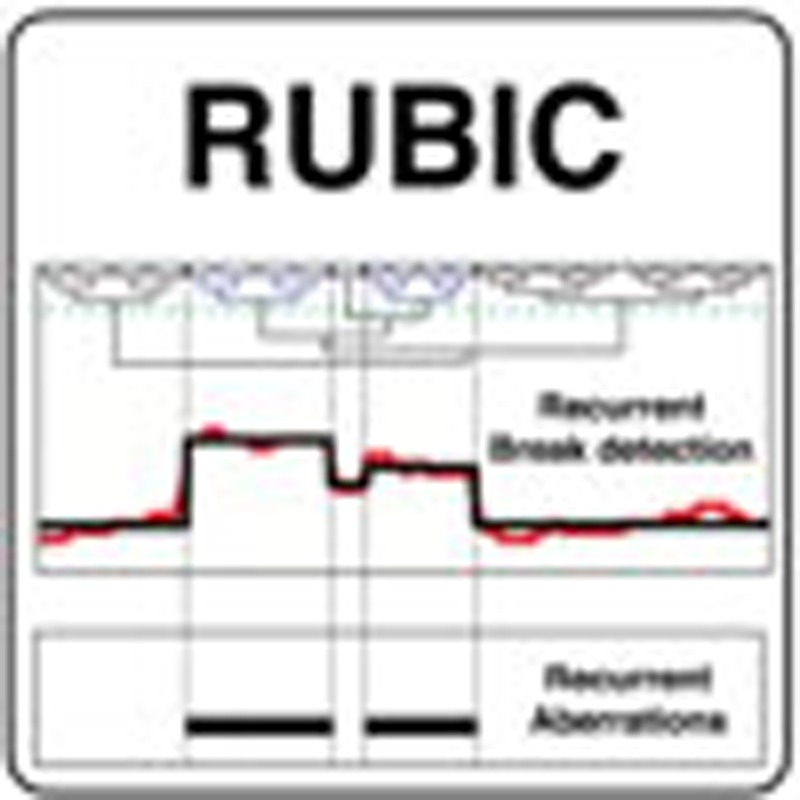

Owing to genomic instability, cancer cells often exhibit a large number of somatic copy number aberrations many of which are believed to play a pivotal role in tumour development or progression. Specifically, somatic copy number aberrations represent one of the mechanisms to activate oncogenes and inactivate tumour suppressors^1^,^2^.

Given a large collection of somatic copy number profiles of tumours, an important challenge is to distinguish driver from passenger aberrations. The exact genomic locations of somatic passenger aberrations are expected to be variable across different tumour samples. In contrast, driver aberrations often recur on the same locus across tumour samples, which allows them to be identified in a properly defined statistical framework. Identification of driver aberrations is important as it allows us to identify (new) oncogenes and tumour suppressors.

Many algorithms have been developed for detecting recurrent copy number aberrations^3^,^4^,^5^,^6^,^7^,^8^,^9^,^10^,^11^,^12^,^13^,^14^, highlighting the relevance of discovering novel oncogenes and tumour suppressors. However, this problem is still far from being solved as state-of-the-art approaches fail to identify known oncogenes and tumour suppressors in large sample sets. For example, while *EGFR* is one of the most frequently amplified oncogenes in Glioblastoma^15^, neither RAIG nor GISTIC2 detects the complete recurrently amplified region harbouring *EGFR*.

One of the main difficulties in detecting recurrent copy number aberrations arises from the heterogeneous nature of driver aberrations across samples, ranging from focal aberrations covering a single gene to broad aberrations spanning a whole-chromosome arm. Algorithms should call recurrent regions as focally as possible to pinpoint the driver genes and hence maximizing specificity. Conversely, too much emphasis on focality could result in driver genes being confused with passengers in close proximity, simply due to off-target focal passenger aberrations overlapping with a broader recurrent locus. This results in reduced sensitivity. Therefore, a proper approach should strike a good balance between sensitivity and specificity.

The great majority of algorithms, including the algorithm we propose, start by splitting copy number gains and losses into separate data sets and therefore detect oncogenes and tumour suppressors separately. Throughout, we will only consider the copy number gains—deletions are treated in a symmetric fashion. When considering gains, the first step of existing algorithms is to detect broad loci that are amplified at a significant frequency. Subsequently, heuristics are applied to identify separate focal recurrences within these loci (Fig. 1a–g). This so-called peak splitting is achieved in two possible ways. In the first approach, the null model is adapted^12^,^16^ based on the local background to determine whether a smaller locus is recurrently amplified in an already recurrent locus. This requires the re-estimation of many parameters on smaller loci, resulting in a loss of statistical power. The second approach employs greedy peel-off algorithms^5^,^6^,^11^ that call local maximum peaks in recurrent loci (Fig. 1b,c) and then remove all aberrated segments that overlap with the identified maximum peak (Fig. 1d). Subsequently, new maximal peaks are identified (Fig. 1e) based on a reduced data set, and this loss in power can result in potentially missing important driver genes in close proximity to the original maximum peak. After iterating these steps, a list of independent peaks are generated (Fig. 1f). The boundaries of these peaks are sensitive to passenger aberrations and a post processing step is employed (for example, the RegBounder algorithm^6^) to broaden the peaks and improve the probability of including the correct driver genes (Fig. 1g).

With Recurrent Unidirectional Break Identification by Clustering (RUBIC) we follow a completely different approach. Specifically, RUBIC detects recurrent copy number breaks instead of recurrent amplifications or deletions. A recurrent break marks a region where a significant portion of the samples show transitions in copy number from neutral to gain (positive break) or from gain to neutral (negative break; Fig. 1i). RUBIC is based on a simple idea: if we can prove significant recurrence of breaks that occur in close proximity of each other, a subset of these breaks are most likely associated with driver aberrations. Regions enclosed between recurrent positive breaks on the left and recurrent negative breaks on the right will most likely harbour a putative oncogene. This new approach has several advantages. First, it simplifies the identification of recurrent regions significantly: there is no need for complicated peak splitting or peel-off algorithms. Second, power is maximized as the recurrent breaks are identified based on all samples and by employing a null model based on the behaviour of passenger aberrations on the complete genome. This is in contrast to peak splitting approaches that require recursive re-estimation of the null model on an ever-decreasing locus width or recursive identification of maximal peaks on an ever-decreasing number of samples in peel-off algorithms.

In summary, by focusing on recurrent breaks, RUBIC becomes independent of the regions between the breaks. Specifically, RUBIC circumvents the difficulties of current algorithms outlined above which stem from aiming to call regions at the right size. RUBIC is simple, computationally efficient and outperforms existing methods on both simulated and real data sets. It calls more true-positive regions (between 1.4 and 3.6 times more than GISTIC2) at more (appropriate) focal widths thus pinpointing the responsible driver genes. Finally, the algorithm only requires a single parameter, controlling the false discovery rate (FDR) of called regions.

## Results

### Overview

**Figure 1:**
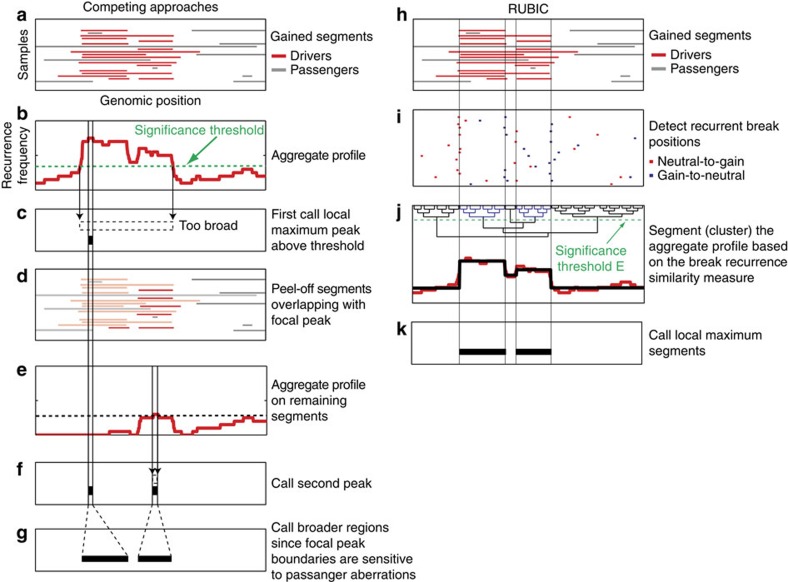
Algorithmic steps of competing approaches and RUBIC. (**a**) A heat map of the gains in simulated copy number profiles for 20 samples. Segments that activate oncogenes (driver aberrations) are shown in red and passenger aberrations in grey. (**b**) The copy number profiles in **a** are aggregated (summed) to produce the aggregate gain profile. The dashed line represents a significance threshold based on a null model, obtained by, for example, permutation of the probe indices in **a**. (**c**) The calling of the maximum peak in the aggregate profile within the genomic region where the aggregate profile exceeds the significance threshold. (**d**) Copy number segments overlapping with the maximal peak are removed from the data set. (**e**) Based on the adapted data set, a new aggregate profile and significance threshold are computed. (**f**) As in **c**, a maximum peak is called in the adjusted aggregate profile. (**g**) Finally, a post processing step is employed to broaden the peaks and improve the probability of including the correct driver genes. (**h**) The same input data set depicted in **a**. (**i**) Positions of recurrent breaks in the copy number profiles. Neutral-to-gain breaks are depicted in red and gain-to-neutral breaks in blue. (**j**) The segmented profile (in black) resulting from performing hierarchical clustering on the aggregate profile (in red). During clustering, RUBIC employs the expected Euler characteristic as similarity measure, thus allowing termination of the clustering when all segments are separated by significant breaks with similarity measures below a fixed, predetermined threshold (green dashed line). The dendrogram resulting from clustering the aggregate profile is also depicted, with the significance threshold used as cutoff to produce the depicted segmentation. (**k**) Local maximum segments are called and such segments are expected to contain putative oncogenes.

RUBIC detects significantly recurrent breaks in the aggregate copy number profile of a collection of tumour samples (Fig. 1h–k). Essentially, RUBIC performs hierarchical clustering on the aggregate profile (Fig. 1j, red line). It starts with segments spanning a single-measurement probe and iteratively joins neighbouring segments until a significant break between segments in the aggregate profile is encountered. As only neigbouring segments can be joined, the complexity of the clustering problem is significantly reduced. Owing to the nature of hierarchical clustering, this implies that all remaining breaks between segments in the aggregate profile are significant. All significant breaks in the aggregate profile represent segment boundaries, and the average aggregate copy number profile between breaks represents the segment amplitude (Fig. 1j, black line). To determine the significance of a break, we require a break recurrence measure and a significance test. The break recurrence measure, which scores a break between two adjacent segments, is equal to the difference in segment amplitudes. Intuitively, this makes sense, since a high frequency in breaks (across samples) results in a large jump in the aggregate (Methods section). Significance of the recurrence measure is represented by the expected Euler characteristic (Methods section), and we employ a null model obtained through cyclic permutation of the tumour profiles (Methods section). During the hierarchical clustering, RUBIC employs the expected Euler characteristic as similarity measure, thus allowing termination of the clustering when all segments are separated by significant breaks with similarity measures below a fixed, predetermined threshold, *E*. We choose to use the expected Euler characteristic as a significance measure because it directly links the global threshold, *E* (used to terminate clustering) to the expected number of false-positive regions called in Fig. 1k (Methods section). This results in error control at the segment level, rather than the probe level, as in competing approaches. The clustering produces a segmented aggregate profile, where the positions of the breaks in the aggregate profile indicate regions of significantly recurrent breaks in the sample profiles (Fig. 1j). Finally local maximal segments are called (Fig. 1k). Such segments are expected to contain putative oncogenes as only gains were employed in this example. Our implementation of RUBIC can be downloaded at http://ccb.nki.nl/software/.

### Benchmarking on simulated data sets

To benchmark RUBIC and competing approaches, we generated a simulated data set of copy number profiles. In contrast to most available simulation approaches that artificially insert recurrent copy number aberrations of fixed widths at any given locus, we employed a preselected set of 100 driver genes as starting point. We generated a copy number profile for each sample based on an idealized evolutionary model. Briefly, we simulate genomic instability by inserting random amplifications and deletions across the genome for many individual cells. In some cells, amplifications activate oncogenes and deletions inactivate tumour suppressors. Such driver aberrations modulate the proliferation rate of an individual cell. The cell with the highest score is then regarded as the dominant clone which we use to represent the sample. This process is repeated for each sample in our analysis. Simulated copy number profiles exhibit complex recurrence patterns developing on both focal and broad scales. For more information on the model and the simulated profiles see the Methods section.

We systematically compared RUBIC with GISTIC2 (a state-of-the-art approach) and RAIG (a recently proposed approach) on simulated data sets generated using our evolutionary model. We employed all three algorithms to separately detect recurrent amplifications and deletions.

For GISTIC2 and RAIG we used exactly the same parameter settings as for the real tumour data sets (Supplementary Methods) RUBIC requires only a single parameter to be set: the FDR. For all algorithms, results were generated at an FDR level of 25%. Each algorithm reports a list of regions and genes (partially) overlapping with these regions. We removed all called regions that did not overlap with any genes. Such regions were never reported by RUBIC or RAIG. Only GISTIC2 reported four such regions in all simulations performed, and suggested nearby genes in brackets, none of which were drivers. We also removed regions >10 mega base pairs (Mbp), since they usually contain many genes and that makes it difficult to pinpoint the drivers. Although rare, such broad regions are sometimes called by GISTIC2 and RUBIC, but not RAIG.

We evaluated the performance based on three measures: (1) the proportion of driver genes that overlapped with called recurrent regions (true positives); (2) the proportion of called regions that do not overlap with any of the driver genes (false positives) and (3) the average driver density in called regions. The third measure scores the ability of algorithms to call regions as focally as possible, that is, the capacity to pinpoint drivers.

**Figure 2:**
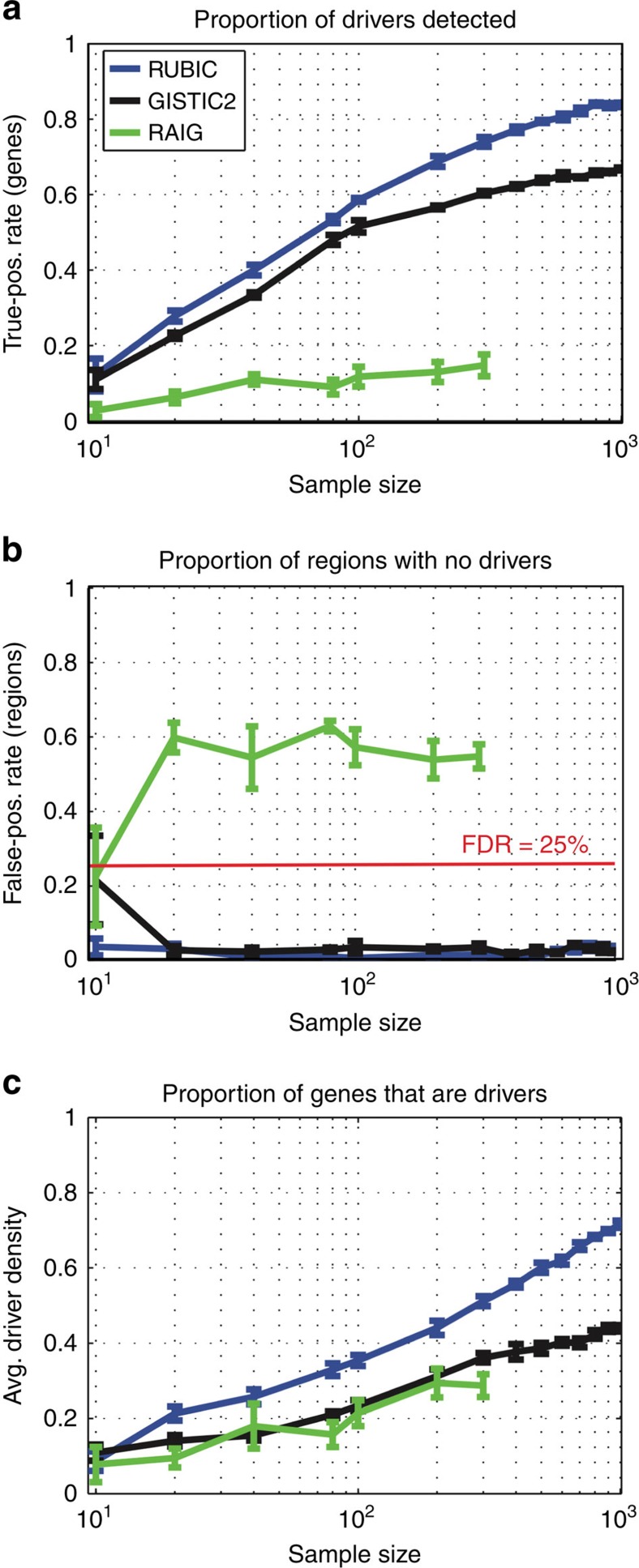
Performance on simulated data. The performance of RUBIC, GISTIC2 and RAIG at recovering driver genes in simulated data for different sample sizes (represented on the *x* axes). (**a**) Proportion of (known) driver genes that overlapped with called recurrent regions. (**b**) Proportion of called recurrent regions that overlapped with none of the driver genes. (**c**) Average (across called regions) proportion of genes that are drivers within each called recurrent region. Error bars represent the s.e. based on five samplings.

We varied the number of samples from 10 to 1,000, and for each number of samples we generated five simulated data sets from which we extracted recurrent regions. RUBIC outperforms both GISTIC2 and RAIG in terms of the number of drivers detected as well as the driver density while controlling the FDR (Fig. 2). Both RUBIC and GISTIC2 achieve an FDR well below the set rate of 25%. For RUBIC, the measured false discovery rate is stable at 5% across sample sizes, but much lower than the 25% FDR selected. This is due to the fact that the cyclic shift null model is conservative. Even though RAIG performs fairly well on previously reported simulation studies, it performs significantly worse than RUBIC and GISTIC2 on all measures for this evolutionary model. In addition, RAIG does not scale well computationally with regard to the number of samples. In Fig. 2 we only show RAIG results for up to 300 samples as we only depict results for processes that took <2 weeks to complete.

### Comparison on three TCGA SNP6 data sets

We compared RUBIC, RAIG and GISTIC2 on SNP6 copy number profiles of three cancer data sets from The Cancer Genome Atlas (TCGA): 1,080 breast invasive carcinoma (BRCA) samples, 577 glioblastoma multiforme (GBM) samples and 450 colon adenocarcinoma (COAD) samples. We used optimized parameters for GISTIC2 and RAIG as specified in the Supplementary Methods. We set the FDR at 25% and extracted both recurrent amplifications and deletions with all three algorithms. As in the simulation experiments, we only report regions that overlapped with at least one gene and excluded all regions >10 Mbp.

Unlike the simulation study, we do not know which genes are drivers and therefore we selected 463 genes (Supplementary Data 1) as positive controls from the Sanger Institute Cancer Gene Census (referred to as ‘Census genes')^17^. We score each algorithm based on four measures: (1) the total number of focal recurrent regions detected (‘no. regions'); (2) the number of regions that overlap with Census genes (‘no. Census regions'); (3) the total number of Census genes detected (‘no. Census genes') and (4) the average driver density in the called regions. The driver density of a region is defined as one divided by the number of genes overlapping the region and is therefore a measure of how good the respective algorithms are at identifying drivers. While this (conservative) measure is optimal when every region contains a single driver, we do not rule out the possibility of multiple weak drivers in a region. If the data supports the presence of multiple (weak) drivers, as suggested in the literature^18^, RUBIC will detect these. Table 1 summarizes the results obtained for the three algorithms on all three data sets. Each entry has two values (separated with a slash) representing recurrent gains and losses, respectively.

RUBIC calls more recurrent regions than both GISTIC2 and RAIG on all three data sets (Supplementary Data 2–7). Given that the FDR was set at 25%, the majority of these regions are expected to contain true driver genes. In fact, the larger number of recurrent regions called by RUBIC also results in a larger yield of Census genes. RAIG calls fewer amplified regions than GISTIC2 on the BRCA data set, but none of these regions contain a Census gene. On the GBM and COAD data sets, RAIG calls more regions than GISTIC2; however, the called regions contain fewer Census genes. These results suggest that the RAIG error rate is high, which is consistent with our observations in the simulation study. The superior ability of RUBIC and GISTIC2 to recover Census genes was also confirmed by a global analysis. Specifically, by employing a cyclic permutation test, we found an enrichment for Census genes (*P*<0.05, permutation test) in all data sets for both RUBIC and GISTIC2, but not for RAIG. In fact, only the amplified regions called by RAIG on the GBM data set showed significant enrichment for Census genes.

While the average driver density estimates for RUBIC are smaller than those obtained by GISTIC2 for the gains, these values are not strictly comparable since RUBIC calls many more regions. This is because recurrent regions that are only detected by RUBIC do not recur as frequently as those that were detected by both algorithms. Regions of lower recurrence will necessarily be called broader and therefore result in a lower average driver density for RUBIC. Specifically, if we only look at the 27 amplified regions where RUBIC and GISTIC2 overlap in BRCA, the average driver densities are comparable, with 0.29 and 0.35 for RUBIC and GISTIC2, respectively. Of these 27 amplified regions, 16 were called (slightly) more focally by GISTIC2. However, 6 of these regions called by RUBIC included extra Census genes. In contrast, none of the 11 regions that were called more broadly by GISTIC2 included any extra Census genes. These results suggest that GISTIC2 indeed tends to call amplified regions too focally. Perhaps the best example illustrating that GISTIC2 tends to call amplifications too focally is *EGFR* in glioblastoma. The skeptical reader might suspect that we ran GISTIC2 with sub-optimal parameters, but in fact we downloaded the GISTIC2 results (with optimized parameters) from http://firebrowse.org/. Counter-intuitively, if we run GISTIC2 on smaller subsets (<577) of the Glioblastoma data set, we do actually detect *EGFR*. The reason is that GISTIC2 calls regions wider for smaller sample sizes (Fig. 2c), but ironically falls prey to passenger aberrations that ‘distract' from the true driver aberration at larger sample sizes.

Deletions called by RUBIC are more focal than those called by GISTIC2 (higher average driver density in Table 1), while the opposite is true for amplifications. The asymmetry between achieved average driver densities (gains versus losses) in the RUBIC results makes sense from a biological perspective: while tumour suppressors can be inactivated by deletions of sub-genic size, aberrations resulting in overexpression of oncogenes typically cover the whole gene and are therefore expected to be wider. RUBIC only called 8, 15 and 9% of the amplified regions based on a break inside a gene for the BRCA, GBM and COAD data sets, respectively. In contrast, 34, 65 and 53% of all called deletions were based on a break inside a gene for the same respective data sets.

**Figure 3:**
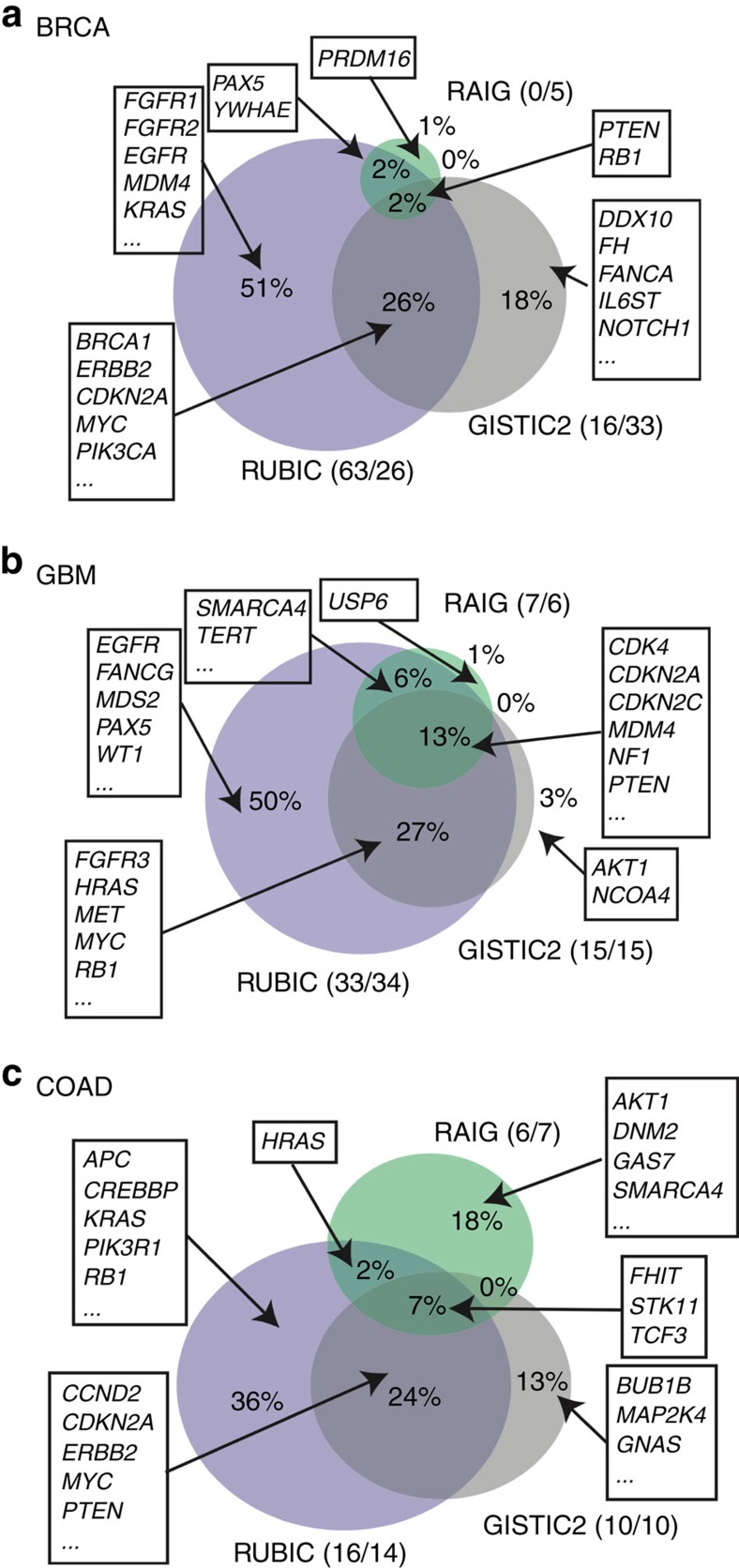
Detected Census genes. Venn diagram of Census genes that overlapped with called recurrent regions in RUBIC, GISTIC2 and RAIG. (**a**–**c**) illustrates this for the breast (BRCA), glioblastoma (GBM) and colon (COAD) cancer data sets, respectively. The numbers separated by a slash (in brackets) represent Census gene counts for gains and losses, separately.

When considering the overlap in the Census genes retrieved by the three approaches (Fig. 3) we notice that RUBIC returns the largest number of Census genes and that the majority of the Census genes retrieved by GISTIC2 and RAIG are a subset of the Census genes retrieved by RUBIC. In the breast cancer data set, GISTIC2 was able to call a single unique broad amplified region that was not detected by RUBIC. This region resides at the end of chromosome 1q and contains a single Census gene. For the deletions, GISTIC2 called 11 unique regions that were not detected by RUBIC. Three of these regions overlapped with Census genes. One of these regions on Chromosome 9q is very broad (9 Mbp) and contains seven Census genes. This single region explains most of the disparity between the results of RUBIC and GISTIC2 in Fig. 3a. RUBIC did call this region, but it was filtered out as it just exceeded 10 Mbp.

**Figure 4:**
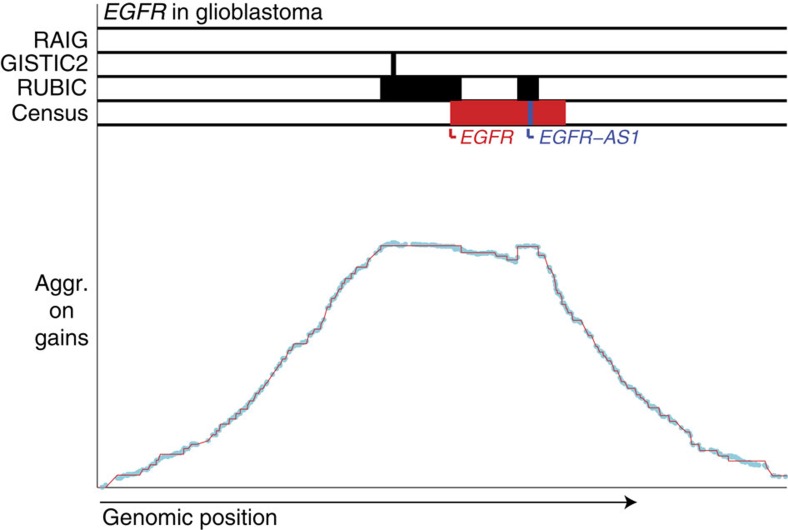
Recurrence at the *EGFR* locus. Genomic representation of *EGFR* and called recurrent regions in its proximity by RUBIC, GISTIC2 and RAIG on the Glioblastoma data set. The cyan profile represents the aggregate copy number profile. The RUBIC segmented aggregate is depicted in red. The rows with labels RUBIC, GISTIC2 and RAIG show the genomic locations of regions called by each of these algorithms. The row with label ‘Census' shows the location of *EGFR* (in red) and *EGFR-AS1* (in blue).

Some known oncogenes and tumour suppressors are only captured by RUBIC, such as *MDM4* in breast, *APC* in colon and *EGFR* in Glioblastoma (Fig. 3). *EGFR* is the most frequently amplified gene in Glioblastoma, yet neither GISTIC2 nor RAIG detects it. GISTIC2 missed *EGFR* because it called a false focal peak (containing no overlapping genes) near *EGFR* and peeled away most of the segments overlapping with the false peak that also overlap with *EGFR* (Fig. 4). Interestingly, RUBIC calls two regions, consistent with the observation that 24–67% of all glioblastoma's are type III deletion mutants where exons 2–7 are deleted^19^. This result also suggests that *EGFR-AS1* might be an oncogene in its own right.

### Focused analysis of the breast cancer data set

**Figure 5:**
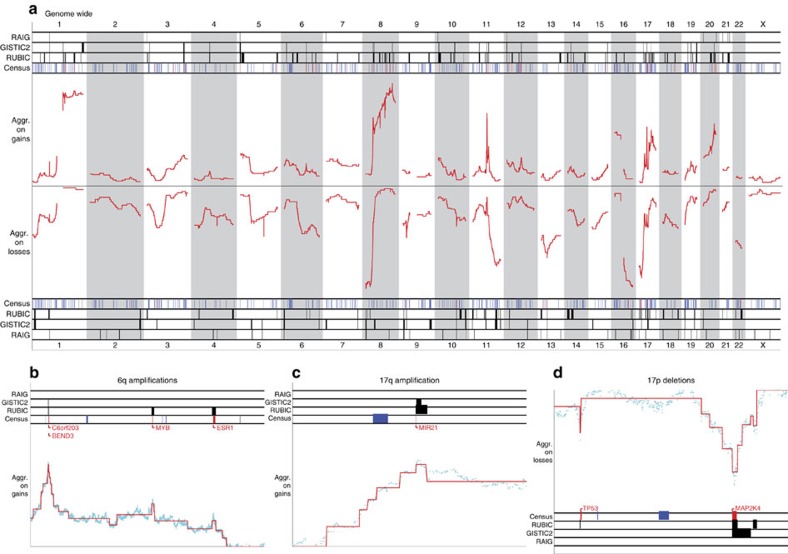
Genome-wide overview of detected regions in breast cancer. Genomic representation of recurrent regions found by RUBIC, GISTIC2 and RAIG in the breast cancer data set. (**a**) RUBIC segmented aggregate profiles (in red) across the whole genome for gains and losses in the top and bottom halves, respectively. The rows with labels RUBIC, GISTIC2 and RAIG show the genomic locations of called recurrent regions. The row with label ‘Census' shows the locations of Census genes in blue. Validated breast cancer genes are represented in red (in the Census row). (**b**–**d**) Example (zoomed in) loci with validated gene names added in red. The cyan profiles represent the aggregate of all samples before RUBIC segmentation depicted in red.

We analysed the BRCA data set more closely and show a genome-wide overview of called regions by all three algorithms in Fig. 5. Here we also highlight (in red) a small subset of bona fide and/or recently validated oncogenes (52 in total) and tumour suppressors (12 in total) specifically associated with breast cancer. The list is constructed based on strong evidence for the involvement of each of the genes in breast cancer, and is largely based on two published lists^16^,^20^. See Supplementary Table 1. A subset of the oncogenes in this list were only recently validated^16^. RAIG, GISTIC2 and RUBIC were able to recover, respectively, 0, 13 and 34 of these bona fide oncogenes and 2, 5 and 5 of these tumour suppressors. All five regions containing the tumour suppressors were called more focally by RUBIC as compared with GISTIC2. A global enrichment test with a cyclic permutation scheme shows that all RUBIC and GISTIC2 regions are highly enriched for bona fide oncogenes and tumour suppressors (all *P* values <10^−3^, permutation test).

Zooming in on some loci, we illustrate examples in which RUBIC outperforms both GISTIC2 and RAIG. First, we find that RUBIC is able to recover four validated oncogenes missed by both GISTIC2 and RAIG (Fig. 5b). In the second example, GISTIC2 called an amplification peak too focally and missed *MIR21* (Fig. 5c). Finally, we show an example where GISTIC2 called a too broad deletion in *MAP2K4* (Fig. 5d). More generally, for regions overlapping with RUBIC, GISTIC2 consistently calls broader deletions without introducing any extra known tumour suppressors or Census genes, while the broader amplifications called by RUBIC do include more oncogenes and Census genes as compared with those called by GISTIC2. This suggests that the smaller deletions and larger amplifications called by RUBIC improve driver detection.

### Comparison on next-generation sequencing

To investigate the applicability of RUBIC to copy number profiles derived with next-generation sequencing (NGS) technology, we compared RUBIC and GISTIC2 on two additional NGS data sets (Supplementary Data 8–11). The first data set consists of copy number profiles of 90 breast cancer samples (not overlapping with TCGA samples) derived from low coverage (<1 × average) whole-genome sequencing (lcWGS)^21^. The second set contains 383 TCGA breast cancer copy number profiles derived from whole-exome sequencing (WES). Since all comparisons indicated that RAIG is not a competitive approach, we only benchmarked RUBIC against GISTIC2 on the NGS data sets. We used optimized parameters for GISTIC2 as before, set the FDR at 25% and extracted both recurrent amplifications and deletions with both algorithms. As before, we only report regions that overlapped with at least one gene and excluded all regions >10 Mbp. The results indicate that both RUBIC and GISTIC2 can be successfully applied to NGS data, as we recover recurrent regions containing known drivers, albeit at a lower average driver density. The lower density is a direct consequence of the fact that the sample size of the NGS data is lower compared with the SNP6 data, resulting in larger called regions and hence a lower average density. The results also indicate that the observations we made based on the SNP6 data regarding the relative performance of RUBIC and GISTIC2 can be extrapolated to NGS data (Table 2). Specifically, we show that RUBIC consistently identifies more recurrent regions, more Census genes and more bona fide breast cancer genes at comparable or higher driver densities. On the lcWGS data set, RUBIC detects a larger number of Census genes, in spite of the fact that the sample size is much lower than the SNP6 breast cancer data set. This is most likely caused by two factors. First, the lcWGS set contains many BRCA-like samples, characterized by BRCA1/2 specific but highly unstable copy number profiles, increasing the likelihood of detecting recurrent aberrations. Second, owing to the smaller sample size, the regions called by RUBIC are larger, hence increasing the likelihood of detecting more Census genes. While the overlap of amplified regions identified on lcWGS profiles with the SNP6 recurrent regions is ∼50%, it is consistent at that level for both RUBIC and GISTIC2. There are two reasons why we expect this overlap to be low. First, the collection of samples used for lcWGS is highly enriched for the BRCA-like samples compared with the TCGA SNP6 data set. Second, the collection of patient samples used for the lcWGS does not overlap with the TCGA data set and the obtained overlap is therefore consistent with an FDR of 25%. In contrast, 87% of the amplifications detected by RUBIC in the WES data set overlap with those found in the SNP6 data set. The patient samples in the WES data set are a subset of those comprising the SNP6 data set and there is no enrichment for any particular subtype (the 383 samples were selected randomly). This suggests that RUBIC is robust against the platform differences, in contrast to GISTIC2 that obtains only 61% overlap.

### Fragile site analysis

Since RUBIC calls recurrent breakpoints, it is reasonable to ask whether we are not simply calling breakpoints at fragile sites. To answer this, we would have to test whether the recurrent regions called by RUBIC are enriched for fragile sites. We employed a published list^22^ of fragile sites and combined that with an unpublished list obtained from the Sanger Institute to construct a list of 127 rare and common fragile sites and performed a permutation-based enrichment test (Supplementary Methods and Supplementary Data 12). We could not find any enrichment for fragile sites in recurrent regions called by RUBIC for either the SNP6, lcWGS or WES profiles in any of the cancer types considered, as indicated in Table 3.

## Discussion

To identify cancer genes residing in recurrently aberrated genomic regions, we follow a completely different approach from current state of the art approaches. Rather than focusing on the recurrence of regions, we introduced RUBIC, an approach that considers the recurrence of breaks. This results in a significant simplification of the algorithm as there is no need for recursive identification of smaller recurrent regions in broader regions via complicated peak splitting approaches. An added advantage of the fact that RUBIC focuses on breaks reflecting the relative change in copy number between segments, rather than the cumulative strength of an aberration across samples, is that the need for an arbitrary reference state is diminished. RUBIC requires only a single input parameter (the FDR) and controls the FDR at the level of regions, rather than probes as most competing approaches. Although users are discouraged from inputting raw unsegmented copy number data into RUBIC, we do expect RUBIC to be less sensitive to the choice of a segmentation algorithm since our theoretical approach does not explicitly require piecewise constant segments. In contrast, algorithms like GISTIC2 that directly peel-off segments when calling peaks will be sensitive to the specific choice of segmentation algorithm. In a comparison with GISTIC2 and RAIG, we show that RUBIC calls significantly more recurrent regions and identifies a much larger fraction of regions containing known cancer genes (from the Cancer Gene Census).

We developed a gene centric simulation model to employ in our benchmarking studies. In this model, we define hypothetical driver genes, simulate genomically unstable copy number profiles and apply evolutionary pressure which results in driver genes being selectively aberrated. We believe this is an improvement over existing simulation approaches as (1) it focuses on genes rather than aberrations; (2) it is an approximation (albeit quite rough) of the evolutionary processes going on in real tumours and (3) it produces recurrence patterns that closely resemble patterns in real data sets. It is therefore suited for revealing shortcomings in existing approaches. For example, RAIG reports a very high recall and precision rate in a previous simulation study^14^ which simulated driver aberrations, rather than driver genes. However, it commits many false positives when calling driver genes in data generated with our simulation model. On simulated data from this model, RUBIC outperforms both GISTIC2 and RAIG on all measures: it finds more driver genes, calls very few regions that do not overlap with driver genes and calls these regions more focally. Nonetheless, RUBIC does tend to call fewer false-positive regions than expected based on the set FDR owing to its conservative null-model. This is because many copy number breaks belong to driver aberrations that are not provably recurrent. Yet we include these breaks in our null model which should ideally only contain breaks associated with passenger aberrations.

On the three TCGA data sets we employed, we selected 463 genes from the Cancer Gene Census to employ as positive controls. It should be noted that one should not expect all of these genes to be involved in tumour development and maintenance specifically in breast cancer, glioblastoma and colon cancer, since they have been found to be somatically mutated in a much broader variety of cancer types. Nor should we expect all of them to be activated or inactivated through copy number aberrations, as most of these genes were identified based on the occurrence of other aberrations, such as point mutations. In fact, in some cases we do not even know the status (oncogene or tumour suppressor) of the genes. We therefore also considered a much smaller subset of bona fide or validated breast cancer oncogenes (*n*=52) and tumour suppressors (*n*=12).

As stated in the introduction, algorithms should strive to accurately pinpoint drivers by calling recurrent regions as focally as possible. On the other hand, as we have shown, too much emphasis on focality results in calling passengers (only) in close proximity to drivers (*EGFR* in glioblastoma being a good example). This problem is threefold. First, it results in the driver genes being missed, reducing the true-positive rate. Second, passenger genes are called erroneously, increasing the false-positive rate. Finally, these erroneously called passengers are often reported as highly significant, since they do occur in highly recurrent regions.

Finally, we have demonstrated that RUBIC is not only applicable to SNP6-derived copy number profiles, but can also successfully be applied to copy number profiles derived from NGS data. We showed that the results obtained in the comparison with GISTIC2 on the SNP6 data also hold for NGS data, both in the setting of copy number profiles derived from low coverage whole-genome sequencing as well as WES.

While it is beyond the scope of this work, the methodology of RUBIC can be applied to other application domains. For example, large-scale projects such as The Encyclopedia of DNA Elements (ENCODE) are generating large amounts of ChipSeq data. Typically these profiles are subjected to peak calling to identify, for example, binding sites of transcription factors or domains characterized by a specific chromatin mark. The segmentation approach we proposed here can be employed to segment ChipSeq profiles to identify binding peaks and domains. Note that this will amount to the application of the segmentation to a single sample. However, as ChipSeq is also being applied in tumour material on a more regular basis, we foresee that RUBIC will also be applied to patient cohorts to detect recurrently occurring peaks or domains.

## Methods

### The break recurrence measure

Suppose we have a genomic region *R* of width *w* and cut it at position *g*_0_ into two regions: *R*_L_ and *R*_R_. Let the widths of these regions be denoted by *w*_L_ and *w*_R_, respectively. Let the average of the aggregate profile for regions *R*_L_ and *R*_R_ be denoted by 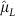 and 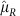, respectively. Positive breaks near *g*_0_, that significantly recur across samples, will result in 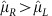. In contrast, under the null model (which models passengers) positive and negative breaks are equally likely to occur anywhere in *R*. From this it follows that, under the null, the expected means will be equal, that is, 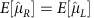. It is important to note that this equality holds even if *R* is fully contained within a recurrent region. It is this observation that removes the need to employ the peak splitting algorithms mentioned in the introduction.

It can be shown that a recurrent break occurs at *g*_0_ in *R* by showing that the value of 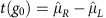 is significant according to the null model. This is similar to performing a two-sample *t*-test where the two samplings are represented by the aggregate log-ratio measurements in *R*_*L*_ and *R*_*R*_ respectively. Formally, *t*(*g*), is also a function of *w*=(*w*_L_, *w*_R_), and will be denoted by *t*_*w*_(*g*). The larger *w*_L_ and *w*_R_, the more statistical power one attains. However, if these regions are too large and extend beyond loci in which recurrent breaks of the opposite sign occur, the power will decrease considerably.

In the Methods section entitled ‘segmentation' we show how hierarchical clustering based on the significance of *t*_*w*_ can be employed to simultaneously find appropriate values for *w*_L_ and *w*_R_ and identify recurrent breaks based on the break recurrence measure, *t*.

### The null model

We employ a null model to describe passenger breaks and hence identify recurrent breaks by evaluating the significance of the break recurrence measure. We use a cyclic shift permutation scheme described in detail in the literature^12^,^13^ to define a null model. To sample from the null distribution, we shift probe indices by a random offset for each copy number profile independently. In this scheme, all break locations become independent across samples, while the inherent genomic dependencies within each sample, for example, chromothripsis patterns, are retained. As with the real data, we sum all cyclically shifted sample profiles per probe (locus) to form one realization of the aggregate profile under the null. (For notational convenience, a specific realization will be denoted by the index *i*.)

By repeatedly permuting profiles one can estimate the probability that breaks recur at observed frequencies by chance alone. This null model is conservative, since we would ideally only model passenger breaks, whereas many of the breaks in our data contribute to driver events. To reduce this bias, we first detect driver breaks with RUBIC and then update the null model after deleting these breaks. We repeat these two steps iteratively (Supplementary Methods, Supplementary Figs 1 and 2).

### Measuring the significance of break recurrence

For a fixed *w*, each *t*_*w*_ can be associated with a (two-tailed) *P* value derived from the null model. We will, instead, use a different measure of significance called the expected Euler characteristic^12^,^23^,^24^. This measure is more natural in our application and will allow us to directly control the false discovery rate on called recurrent regions rather than probes, as explained later. The idea is as follows: for any fixed realization of the null model (indexed with *i*), a fixed *w* and a fixed non-negative threshold *t*, we define positive and negative excursion sets: 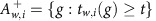 and 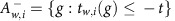, respectively. We count the number of disjoint regions in each and denote these with 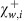 and 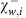, respectively. The sum of these counts, 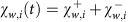, is known as the Euler characteristic. We can then compute the expected Euler characteristic across realizations: 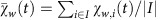, where *I* represents the set of all possible permutations (Supplementary Methods).

On actual data, for a fixed scale *w*_0_ and position *g*_0_, we can compute a value *t*_0_=*t*_*w*0_(*g*_0_). 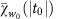 can be interpreted as a measure of significance (small values being significant). In fact, it is an upper bound for the familywise error rate if we regard each locus *g* as a separate test and it is a tight bound for small values: 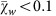 (ref. 23). It is important to note that the Euler characteristic allows us to link the value of the break recurrence measure at a specific locus and a fixed scale, *t*_0_=*t*_*w*0_(*g*_0_), to the significance of the number of called recurrent regions in the aggregate profile.

There are two major advantages of using 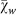 as a significance measure. First, there exists an analytical approximation that relates *t*=*t*_*w*_(*g*) to 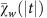 that is highly accurate for the majority of scales (Supplementary Methods, Supplementary Figs 3 and 4). This means that we can avoid time consuming permutation tests for many choices of *w* (Supplementary Methods). The second, and more important reason, is that we can directly compute the false discovery rate on called recurrent regions (not breaks) using 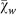. We clarify this in the Methods section describing RUBIC calling.

### Segmentation

Ultimately, RUBIC is a segmentation algorithm on the aggregate profile. We essentially approximate the aggregate profile with a piecewise constant function with jump discontinuities at significantly recurrent breaks. The jump discontinuities represent significant breaks in the aggregate profile. The jump height at position *g* is exactly equal to *t*_*w*_(*g*), where *w*=(*w*_L_,*w*_R_) represents adjacent segment widths. We regard breaks in the aggregate profile as significant if 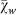 is small.

RUBIC segmentation is an agglomerative hierarchical clustering algorithm that starts with the most fine-grained segmentation, where each probe is a unique segment, and iteratively merges adjacent segments. As a measure of the similarity of two segments, we use 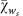, where *w*_s_ corresponds to the widths of the segments under consideration. In each iteration, we merge segments with the highest (least significant) 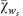 score. We continue merging segments until all remaining 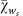 scores are less than or equal to a fixed global threshold, *E*. This implies that the jump discontinuities separating the remaining segments are all significant (<*E*) and hence represent recurrent breaks. In the segmented profile, segments residing between recurrent breaks are represented by a single value, the average of the aggregate profile in that segment. Since all segments are naturally sorted on the genome, and we only need to consider adjacent segments for merging, we can efficiently perform the clustering in *P*log(*P*) time, where *P* is the number of probes on the genome. Figure 1j shows the resulting segmentation when we perform this procedure for a fixed significance threshold, *E*.

### Calling

The final step in the algorithm is to simply call all the local maximum segments in Fig. 1j producing the result illustrated in Fig. 1k. A segment is defined as a local maximum when it is bordered by positive and negative jump discontinuities on its left and right, respectively. One can then expect to find oncogenes inside these called segments since positive (negative) jump discontinuities correspond to significantly recurrent (<*E*) positive (negative) breaks, that is, recurrent amplifications.

The remaining question is: how to choose the global threshold *E*? The benefit of using the Euler characteristic as similarity measure is that *E*/2 is an upper bound on the expected number of false-positive local maximum segments (called regions) that result in the data (Supplementary Methods, Supplementary Figs 5–10). Since there is a direct correspondence between the number of false-positive regions and the threshold *E*, we can directly apply the Benjamini–Hochberg procedure^25^ to control the false discovery rate on recurrently amplified called segments^12^. We illustrate the Benjamini–Hochberg procedure with an example. Suppose we specify the FDR level at 25%. We then start by setting *E*=2 × 0.25, knowing that the expected number of false-positive called regions will be below 0.25. We then count the number of called regions after clustering, say there are 70. At this point we choose *E*=70 × 2 × 0.25. We continue adapting *E* until the number of called regions remain unchanged. Say, for example, we end up with 100 called regions. At this point *E*=100 × 2 × 0.25=50. The expected number of false positives will be below *E*/2=25, which is 25% of the 100 called regions.

### Simulating copy number evolution with known driver genes

Given the lack of a real copy number data sets for which all the oncogenes and tumour suppressors are known, it is very hard to compare algorithms in terms of specificity and sensitivity on real data. This type of analysis can only be achieved through simulation. The majority of simulation studies are performed by artificially inserting numerous recurrent copy number aberrations of fixed widths for any given locus^9^. Such simulations are not designed to give a direct answer to how good algorithms are at pinpointing driver genes, since they define driver aberrations rather than driver genes. In fact, it is questionable whether amplifications of a fixed width recur across multiple samples in real data sets, except for events occurring on the chromosome arm level.

Since real copy number aberrations are subject to selective pressure, we expect oncogenes (tumour suppressors) to be found in recurrently amplified (deleted) regions without the need for fixed recurrent segment widths. For example, an oncogene can be frequently amplified across samples even though the associated aberration widths vary considerably. A small subset of these amplifications might be focal enough so as to cover only this one gene. With enough samples, it is likely that there is a sufficient number of these focal aberrations to unambiguously call the oncogene as being recurrently aberrated. Consequently, finding driver genes is not the same as finding recurrent aberrations of a fixed width.

For that reason, we simulated copy number profiles based on an idealized evolutionary model in which a fixed number of oncogenes and tumour suppressors are known. We assigned a proliferation coefficient to each gene which indicates the influence of that gene on cell proliferation. More specifically, the contribution of a gene to cell proliferation is the product of the coefficient and the average copy number fold change of that gene with respect to the normal diploid state (Supplementary Methods). In our performance study, we selected 100 random genes from the human genome as drivers and assigned to each a proliferation score drawn from a normal distribution. Positive (negative) coefficients represent oncogenes (tumour suppressors). During the simulated evolutionary process, copy number changes were introduced in the profile, resulting in copy number changes in several genes, including oncogenes and tumour suppressors.

We started the simulation by creating a copy number neutral (diploid) dominant clone, with a genome of the same size as the human genome. We then evolved the copy number profile of a single sample by repeating the following randomization and selection steps 20 times:

- Randomization: derive 100 descendants from the dominant clone by adding 10 random copy number aberrations at random locations on the genome. The width and copy number log ratios of the aberrations are extracted from the TCGA breast cancer data set (Supplementary Methods).
- Selection: based on the proliferation coefficients and copy number values of the 100 selected driver genes, compute the overall proliferation of each descendent, select the descendent with the highest proliferation score and define it as the new dominant clone.

The final dominant clone represents the final copy number profile of a single sample. This process is repeated for every sample. Simulated copy number profiles resemble what we observe in real data, with complex recurrence patterns developing on both focal and broad scales.

### Data availability

The lcWGS and simulated DNA copy number data that support the findings of this study are available in GitHub, https://github.com/ewaldvandyk/RUBIC-datasets.git. The TCGA SNP6 and WES data that support the findings of this study are available from TCGA but restrictions apply to the availability of these data, which were used under license for the current study, and so are not publicly available. We provide full details on the TCGA data that we employed as well as the processing steps that were applied to these data to obtain the input profiles employed in our analyses. Hence, after obtaining the data from TCGA under licence our results can be reproduced. All of the remaining data are available within the Article and Supplementary Information files or available from the authors upon request.

## Additional information

**How to cite this article:** van Dyk, E. *et al.* RUBIC identifies driver genes by detecting recurrent DNA copy number breaks. *Nat. Commun.* 7:12159 doi: 10.1038/ncomms12159 (2016).

## Supplementary Material

Supplementary Information Supplementary Figures 1-10, Supplementary Table 1, Supplementary Methods and Supplementary References

Supplementary Data 1 Cancer Gene Census

Supplementary Data 2 Recurrent Amplifications, Breast Cancer (BRCA), SNP6 profiles, RUBIC and GISTIC2

Supplementary Data 3 Recurrent Deletions, Breast Cancer (BRCA), SNP6 profiles, RUBIC and GISTIC2

Supplementary Data 4 Recurrent Amplifications, Glioblastoma Multiforme (GBM), SNP6 profiles, RUBIC and GISTIC2

Supplementary Data 5 Recurrent Deletions, Glioblastoma Multiforme (GBM), SNP6 profiles, RUBIC and GISTIC2

Supplementary Data 6 Recurrent Amplifications, Colon Adenocarcinoma (COAD), SNP6 profiles, RUBIC and GISTIC2

Supplementary Data 7 Recurrent Deletions, Colon Adenocarcinoma (COAD), SNP6 profiles, RUBIC and GISTIC2

Supplementary Data 8 Recurrent Amplifications, Breast Cancer (BRCA), Whole Exome Sequencing (WES), RUBIC and GISTIC2

Supplementary Data 9 Recurrent Deletions, Breast Cancer (BRCA), Whole Exome Sequencing (WES), RUBIC and GISTIC2

Supplementary Data 10 Recurrent Amplifications, Breast Cancer (BRCA), low coverage Whole Genome Sequencing (lcWGS), RUBIC and GISTIC2

Supplementary Data 11 Recurrent Deletions, Breast Cancer (BRCA), low coverage Whole Genome Sequencing (lcWGS), RUBIC and GISTIC2

Supplementary Data 12 Fragile sites employed in the enrichment analysis.

Peer review file

## Figures and Tables

**Table 1:** Summary of detected regions on SNP6 data set.

**Methods**	**RUBIC**	**GISTIC2**	**RAIG**
*Breast cancer (BRCA;* n*=1,080)*			
No. regions (gains/losses)	100/58	28/31	11/41
No. Census regions (gains/losses)	48/16	15/17	0/5
No. Census genes (gains/losses)	63/26	16/33	0/5
Avg. driver density (gains/losses)	0.21/0.41	0.34/0.10	0.80/0.57
			
*Glioblastoma (GBM;* n*=577)*			
No. regions (gains/losses)	40/152	22/36	25/58
No. Census regions (gains/losses)	23/26	14/13	7/6
No. Census genes (gains/losses)	33/34	15/15	7/6
Avg. driver density (gains/losses)	0.29/0.71	0.39/0.19	0.59/0.56
			
*Colon adenocarcinoma* *(COAD;* n*=450)*			
No. regions (gains/losses)	23/72	17/31	27/50
No. Census regions (gains/losses)	11/12	8/9	6/5
No. Census genes (gains/losses)	16/14	10/10	6/7
Avg. driver density (gains/losses)	0.14/0.58	0.21/0.20	0.36/0.46

Recurrent copy number regions predicted by RUBIC, GISTIC2 and RAIG on BRCA, GBM and COAD. For each subtable containing the results of a specific cancer type, the rows represent the following: the first row (labelled ‘no. regions') represents the total number of focal recurrent regions detected by each algorithm. The second row shows the number of regions that overlap with Census genes. The third row represents the total number of Census genes detected. The last row shows the average driver density in the called regions. Each entry has two values (separated with a slash) representing recurrent gains and losses, respectively.

**Table 2:** Summary of detected regions on NGS data sets.

**Methods**	**RUBIC**	**GISTIC2**
*BRCA (lcWGS;* n*=90)*		
No. regions (gains/losses)	80/43	26/29
No. Census regions (gains/losses)	47/10	17/7
No. Census genes (gains/losses)	90/21	25/20
No. bona fide genes (52 oncogenes/12 tumour suppressors)	32/3	10/2
Enrichment *P* values for bona fide genes	2 × 10^−4^/0.022	<1 × 10^−4^/0.083
Avg. driver density (gains/losses)	0.12/0.24	0.09/0.13
Region overlap with SNP6	0.50/0.42	0.42/0.33
		
*BRCA (WES;* n*=383)*		
No. regions (gains/losses)	46/9	13/3
No. Census regions (gains/losses)	32/4	10/1
No. Census genes (gains/losses)	58/14	16/1
No. bona fide genes (52 oncogenes/12 tumour suppressors)	28/2	10/1
Enrichment *P* values for bona fide genes	<1 × 10^−4^/0.018	<1 × 10^−4^/0.021
Avg. driver density (gains/losses)	0.07/0.25	0.08/0.21
Region overlap with SNP6	0.87/0.56	0.61/0.33

Recurrent copy number regions predicted by RUBIC and GISTIC2 for BRCA data sets derived from low coverage whole-genome sequencing (lcWGS) and TCGA WES. For each subtable containing the results of a specific sequencing platform the rows represent the following: the first row (labelled ‘no. regions') represents the total number of focal recurrent regions detected by each algorithm. The second row shows the number of regions that overlap with Census genes. The third row represents the total number of Census genes detected. The fourth row shows the number of BRCA bona fide oncogenes/tumour suppressors detected. The fifth row shows the enrichment *P* values for bona-fide drivers in regions based on a cyclic permutation test. The sixth row shows the average driver density in the called regions. The final row shows the proportion of regions detected in NGS data that overlap with regions found for the SNP6 TCGA data set. Each entry has two values (separated with a slash) representing recurrent gains and losses, respectively.

**Table 3:**
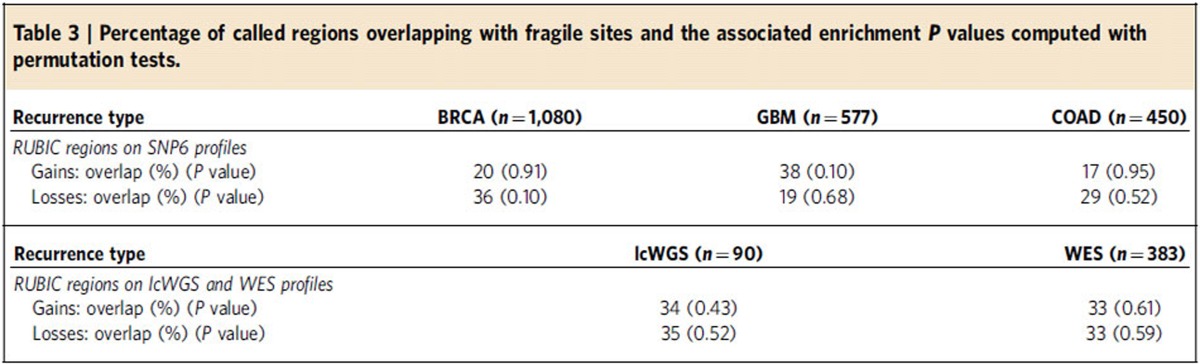
Percentage of called regions overlapping with fragile sites and the associated enrichment *P* values computed with permutation tests.
