# Supplementary material for: RUBIC identifies driver genes by detecting recurrent DNA copy number breaks
Source: Nat Commun. 2016 Jul 11;7:12159. doi: 10.1038/ncomms12159 (PMC4942583; doi:10.1038/ncomms12159)
Supplement: Supplementary Information — Supplementary Figures 1-10, Supplementary Table 1, Supplementary Methods and Supplementary References [file ncomms12159-s1.pdf]

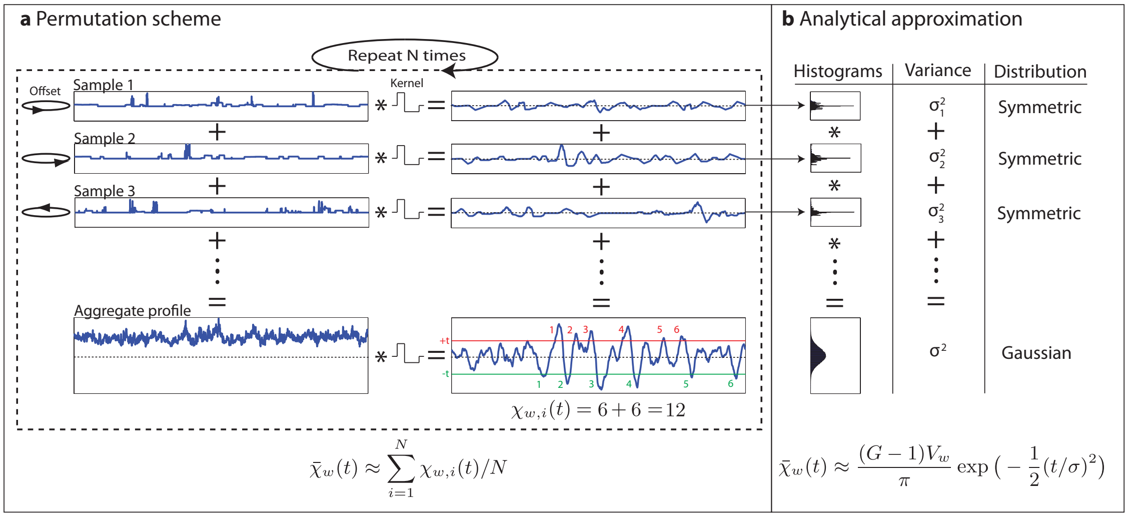

Supplementary Figure 1: **Illustration on how to compute the expected Euler characteristic.** We assume a fixed scale $w = (w_L, w_R)$ and fixed positive threshold t in the null-model. Throughout, $*$ represents the convolution operator. (**a**) Illustration on how to approximate the expected Euler characteristic with the permutation scheme. In one realization of the null, we cyclicly shift each sample's probes with a random offset (independently for each sample). Each sample is convolved with the wavelet kernel corresponding to $w = (w_L, w_R)$ and the results are shown in the curves in the column on the right in **a**. We sum the resulting convolved profiles to obtain the curve in the lower right corner in **a**. Equivalently, we can first sum all the cyclicly shifted samples to produce a realization of the aggregate in the lower left corner and then convolve it with the wavelet. The Euler characteristic is computed by counting disjoint regions above (below) the threshold t ($-t$). This procedure is repeated N times and estimates the expected Euler characteristic using the equation at the bottom of **a**. (**b**) We can accurately approximate the expected Euler characteristic analytically without the permutation scheme if w_L and w_R are large enough. We compute the histogram of each sample's convolved profile. We compute the variance in each histogram and note that they are symmetric. Since all samples are cyclicly shifted independently, we can compute the distribution (variance) on the aggregate by convolving (adding) the histograms (single sample variances). Finally, we approximate the null as a Gaussian process with an analytically derived expected Euler characteristic as shown by the bottom equation in **b**.

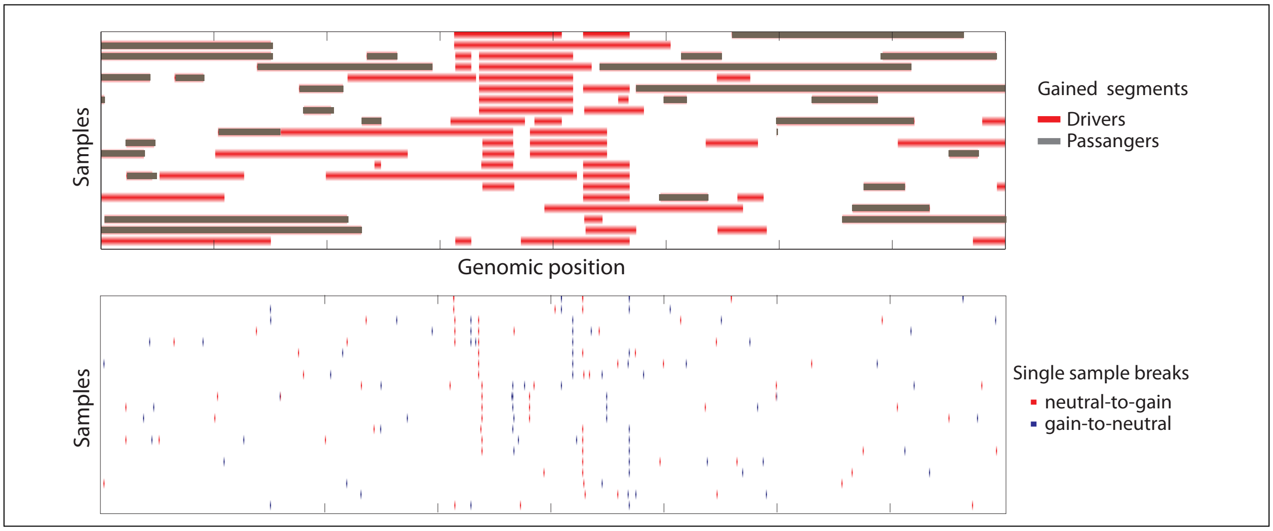

Supplementary Figure 2: **Illustrating an example with 20 DNA copy number profiles.** The top panel shows copy number gains as they occur across the genome (x-axis) for each sample (y-axis). Driver aberrations are shown in red and passengers in grey. The bottom panel shows the copy number break points for each sample. Neutral-to-gain breaks are shown in red, while gain-to-neutral breaks are shown in blue.

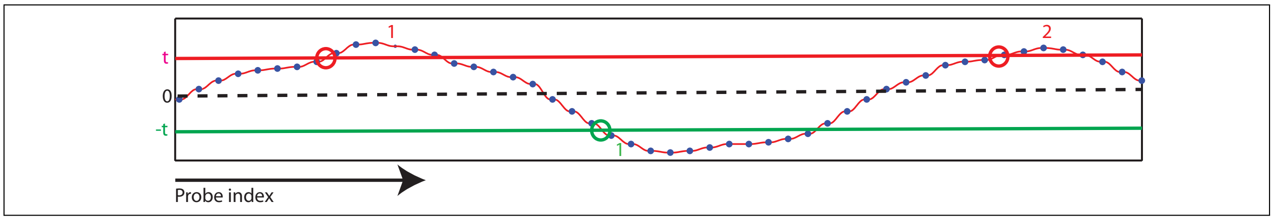

Supplementary Figure 3: **Smooth approximation of a discrete Gaussian process.** We interpolate a discrete stationary Gaussian process (indicated with blue dots) with a smooth and non-stationary Gaussian process (in red). Counting regions above (below) a fixed threshold t ($-t$) can be accomplished by counting up-crossing (down-crossings) in the smoothed profile as indicated by the red (green) circles.

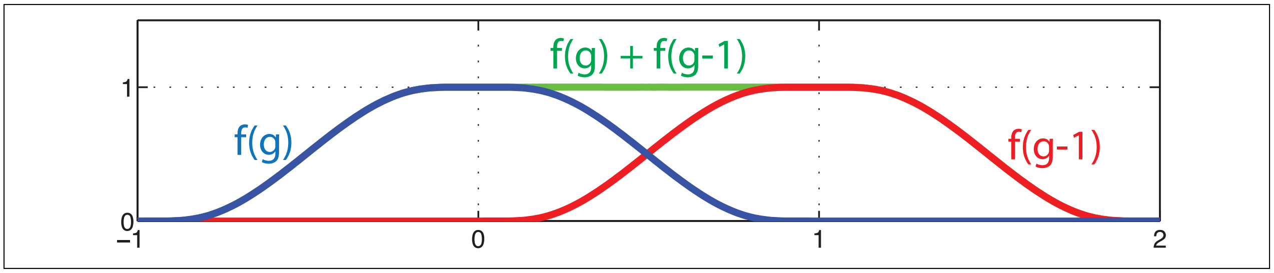

Supplementary Figure 4: **Illustrating the properties of the Bump function used for smoothing the discrete Gaussian process.**

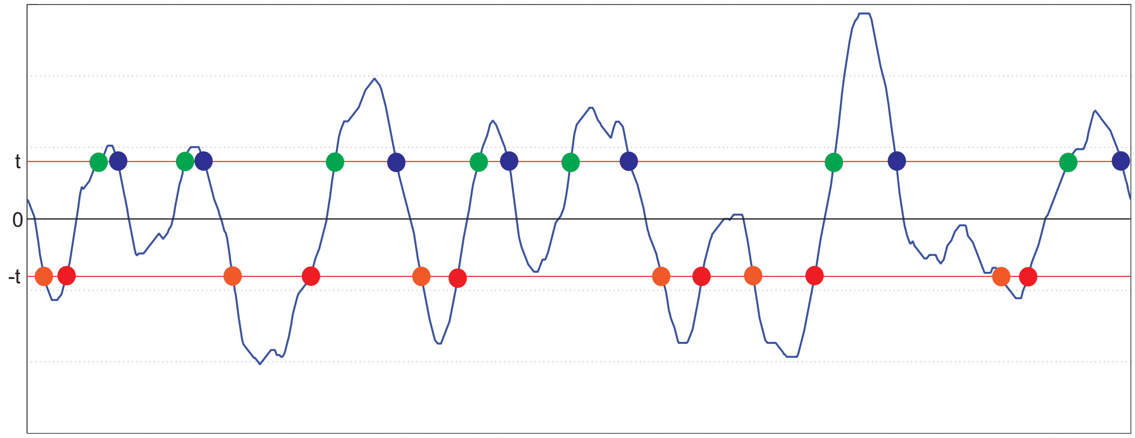

Supplementary Figure 5: **Illustrating the type of t-level crossings that exist.** t up-crossings and down-crossings are represented by green and blue circles respectively. $-t$ up-crossings and down-crossings are represented by red and orange circles respectively

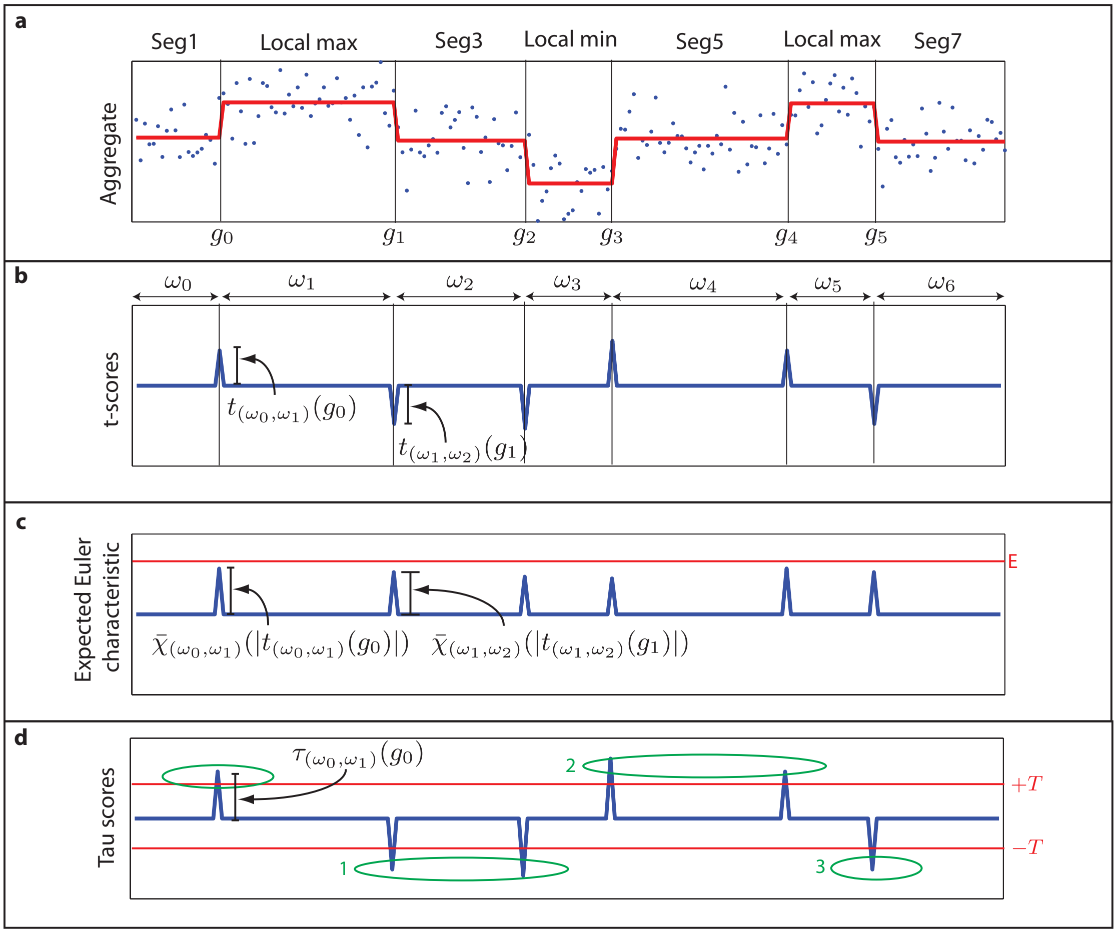

Supplementary Figure 6: **Illustrating how we count local maximum segments.** (**a**) Illustration of the aggregate profile (in blue) and the segmented profile (in red). (**b**) The size of the jump discontinuities in the segmented profile is represented with t-scores. (**c**) Each t-score is transformed into a significance score known as the expected Euler characteristic. (**d**) Each significance score is finally converted to a signed tau-score)

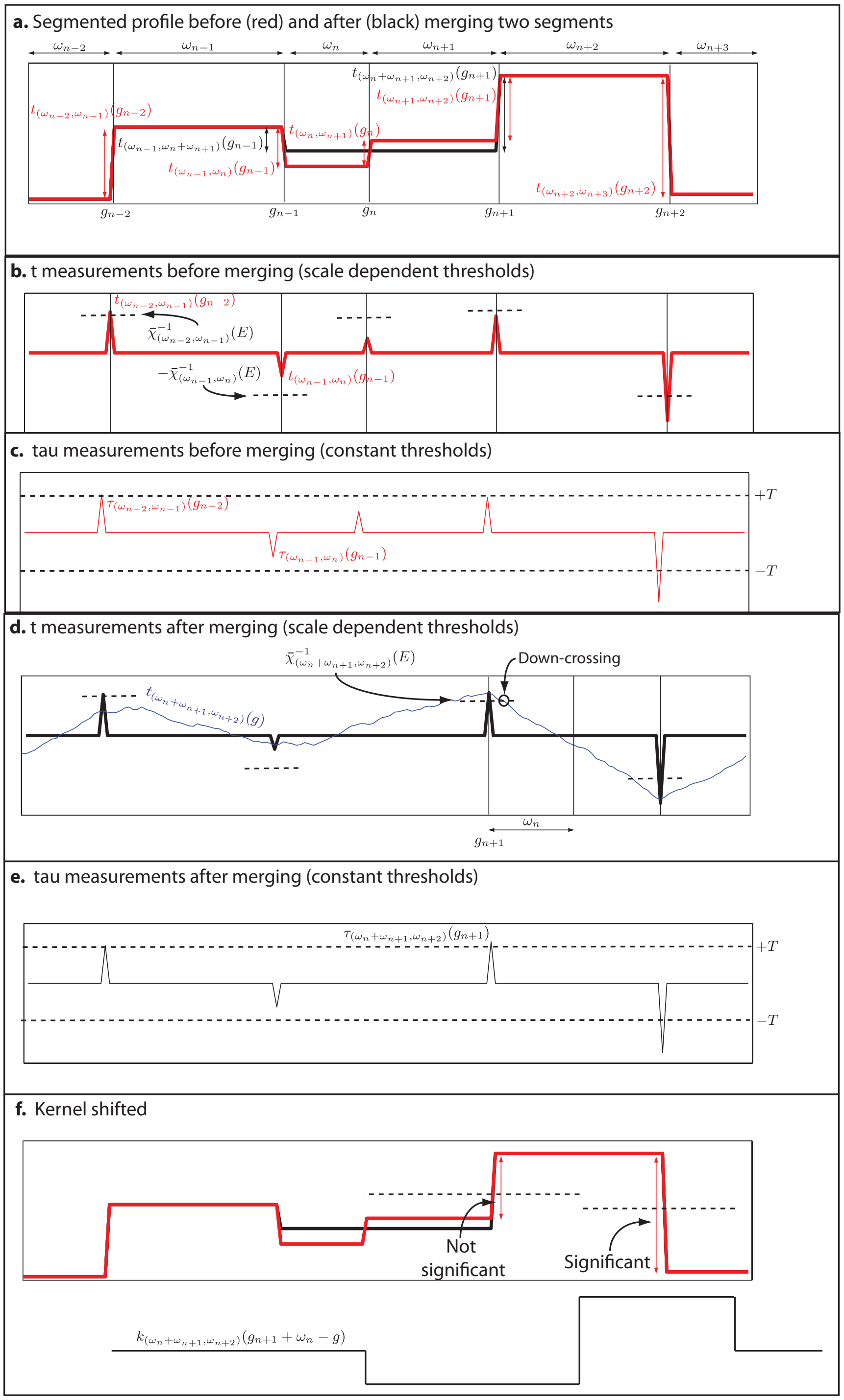

Supplementary Figure 7: **Merging segments in one iteration of clustering.** (**a**) Illustrating the segmented profile before and after merging two segments. (**b**) and (**c**) illustrates the t- (lag-one difference) and tau-scores respectively before merging. (**d**) and (**e**) are the same as (**b**) and (**c**) respectively after merging. In (**d**), we also shows what the aggregate profile looks like after convolving with a fixed kernel. (**f**) We show why the t-score drops below the significance threshold when we shift the kernel.

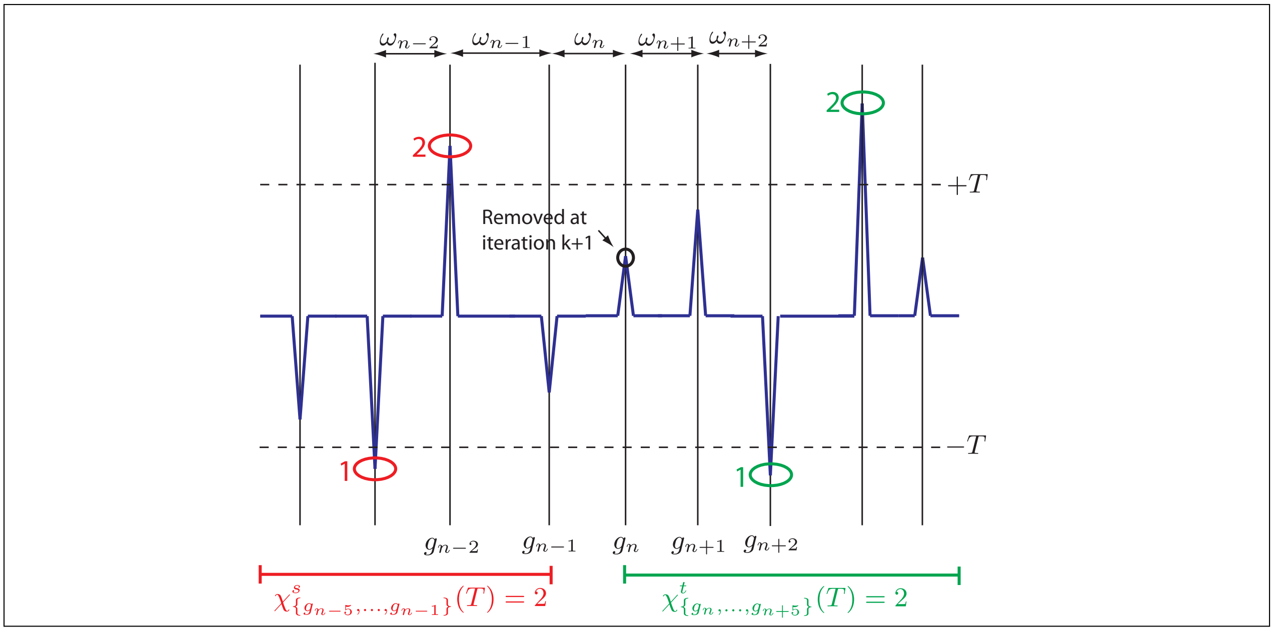

Supplementary Figure 8: **Illustrating Scenario one when merging adjacent segments.** When two segments are merged the encircled break at g_n is removed. This depicts the scenario when the tau-score at break g_{n-2} is above the threshold T and at break g_{n+2} is below $-T$.

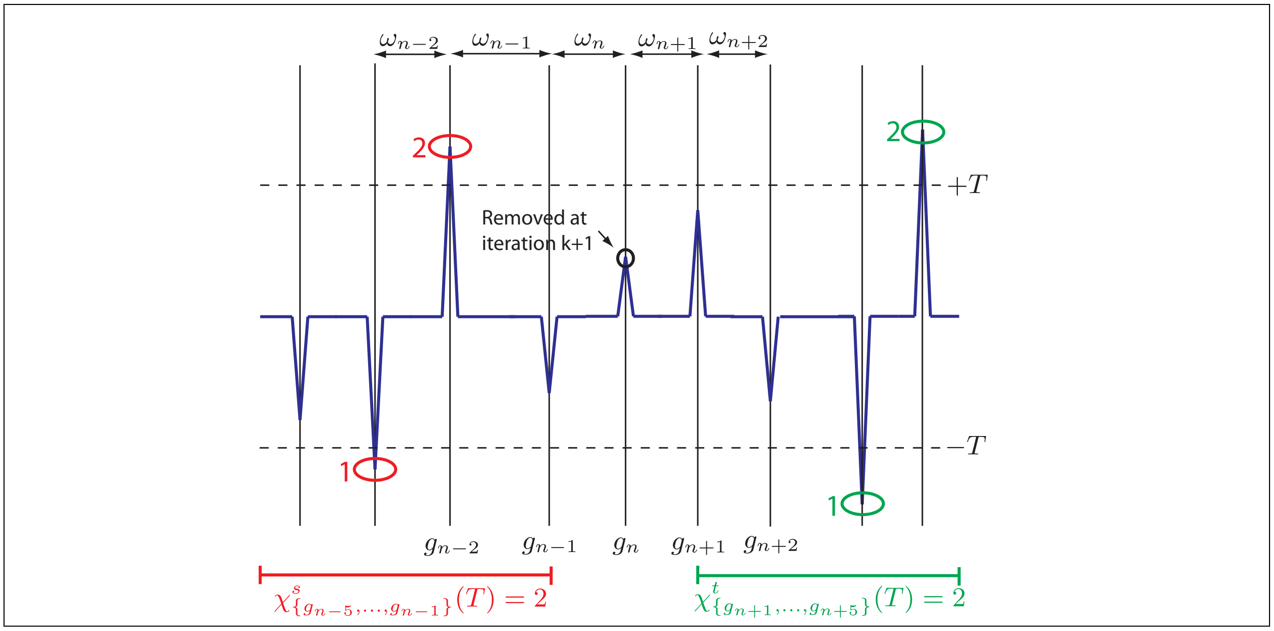

Supplementary Figure 9: **Illustrating Scenario two when merging adjacent segments.** When two segments are merged the encircled break at g_n is removed. This depicts the scenario when the tau-score at break g_{n-2} is above the threshold T, insignificant at g_{n+2} and below $-T$ at break g_{n+3}.

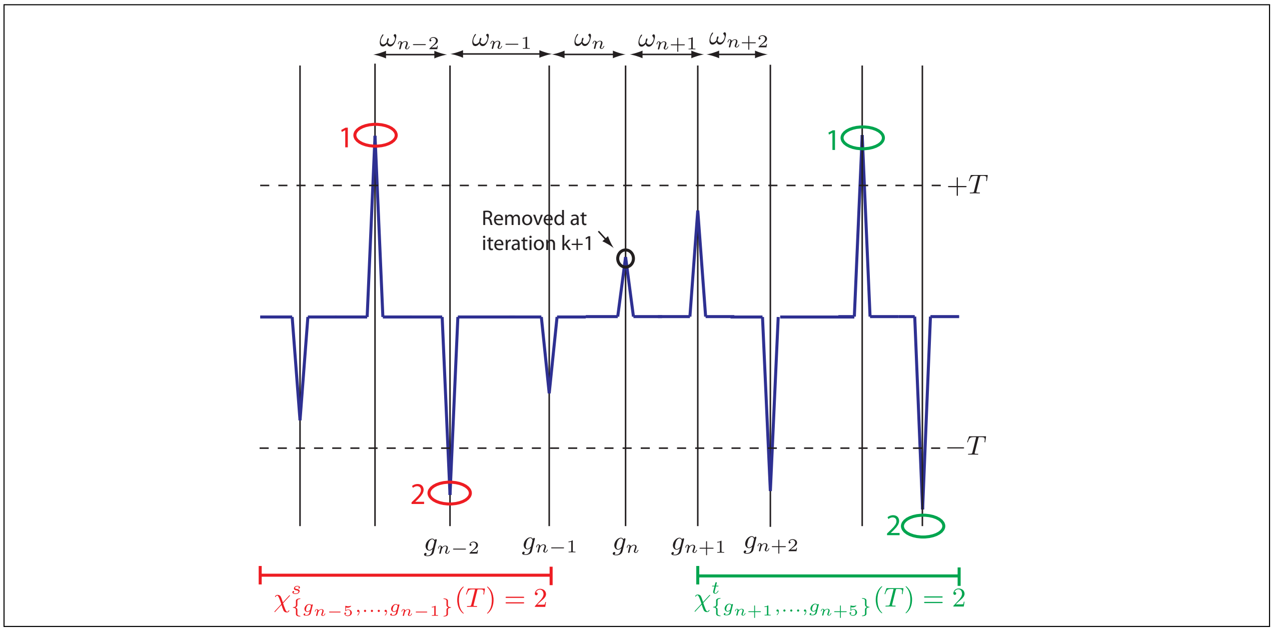

Supplementary Figure 10: **Illustrating Scenario three when merging adjacent segments.** When two segments are merged the encircled break at g_n is removed. This depicts the scenario when the tau-scores at breaks g_{n-2} and g_{n+2} are below $-T$.

Supplementary Table 1: **Bona fide breast cancer genes with the associated literature references.** OG = Oncogene; TS = Tumor suppressor

Gene symbol	Type	Reference	Gene symbol	Type	Reference
AKT1	OG	[1]	AKT2	OG	[2]
AURKA	OG	[3]	BAG4	OG	[3]
BCL2	OG	[4]	BEND3	OG	[5]
C6orf203	OG	[5]	C8orf4	OG	[3]
CCND1	OG	[3,5]	CCNE1	OG	[3]
CDK4	OG	[3]	CKS1B	OG	[3]
CTNNB1	OG	[6]	EGFR	OG	[7]
C11orf30	OG	[3]	ERBB2	OG	[3,5]
ESR1	OG	[8]	ETV6	OG	[9]
FGFR1	OG	[3]	FGFR2	OG	[5]
FGFR4	OG	[10]	GATA3	OG	[11]
GRB7	OG	[3]	IGF1R	OG	[5]
KRAS	OG	[12]	LSM1	OG	[3]
MDM2	OG	[3]	MDM4	OG	[13]
MIR21	OG	[5]	MTDH	OG	[3]
MYB	OG	[14]	MYC	OG	[3,5]
NCOA3	OG	[3]	NIT1	OG	[5]
PAK1	OG	[3]	PIK3CA	OG	[15]
PLA2G10	OG	[3]	PPM1D	OG	[3]
PTK6	OG	[3]	PVRL4	OG	[5]
RPS6KB1	OG	[3]	RSF1	OG	[5]
RUVBL1	OG	[3]	SHC1	OG	[3]
STARD3	OG	[3]	TERT	OG	[16]
TRPS1	OG	[5]	YEATS4	OG	[5]
YWHAB	OG	[3]	ZNF217	OG	[3,5]
ZNF652	OG	[5]	ZNF703	OG	[17]
BAP1	TS	[18]	BRCA1	TS	[19]
BRCA2	TS	[19]	CASP8	TS	[20]
CDH1	TS	[21]	CDKN2A	TS	[22]
EP300	TS	[23]	MAP2K4	TS	[24]
PBRM1	TS	[25]	PTEN	TS	[26]
RB1	TS	[27]	TP53	TS	[28]

Supplementary Methods

Computing the expected Euler characteristic in RUBIC segmentation

Overview. RUBIC segments the aggregate copy number profile using agglomerative clustering. It starts by considering each probe to be a unique segment and continues merging adjacent segments until a stopping criteria is met. For each pair of adjacent segments we compute a similarity measure called the expected Euler characteristic and in each step we merge adjacent segments with the highest similarity measure. This process continues until all similarity measures are below a fixed threshold E. In this section we describe in detail how to estimate the expected Euler characteristic between adjacent segments (which generally depends on the widths of the segments). First, we describe a method involving a permutation scheme that is accurate but is computationally inefficient. Second, we describe an analytical approximation that is often (but not always) accurate and otherwise conservative. Finally, when clustering it is important to decide which approximation to use. We discuss this in the final part of this section. A derivation of the analytical approximation and the reason why the expected number of local maximum segments in the null model will be below the threshold E is discussed in the last two sections of the Supplementary Methods.

Estimating the expected Euler characteristic with permutations. In Supplementary Fig. 1**a**, we give a detailed breakdown on exactly how to estimate the expected Euler characteristic with a permutation scheme for a fixed scale $w = (w_L, w_R)$ and fixed non-negative threshold t.

In one realization of the null (illustrated by the dashed box in **a**), we shift probe indices by a random offset for each sample independently. Probes that are shifted beyond chromosome boundaries are shifted into adjacent chromosomes' start positions and probes shifted beyond the end of the last chromosome are shifted into the start positions of the first chromosome. In this scheme, all break locations are independent between samples, while the inherent genomic dependancies (for example chromothripsis) between breaks are retained within each sample. The next step is to simply sum all the cycled samples' profiles.

To compute the Euler characteristic for a fixed realization, we need to compute $t_w(g)$ at every genomic position g. To efficiently do this we define a wavelet kernel as follows:

$$k_w(g) = \begin{cases} +1/w_R & -w_R \leq g < 0 \\ -1/w_L & 0 \leq g \leq w_L \\ 0 & \text{elsewhere} \end{cases} \tag{1}$$

$t_w(g)$ is computed using this kernel with a computationally efficient operation called convolution (indicated by the symbol $*$ in Supplementary Fig. 1). Essentially, for each locus g_0, it computes $t_w(g_0)$ by 1) reversing and shifting the kernel to location g_0, $k_w(g_0 - g)$, and 2) multiplying it with the aggregate profile (using the scalar product). The resulting convolved profile is shown in the lower right of 1**a**. This is equivalent to first convolving the individual samples' profiles with the

kernel and then summing (because convolution is distributive over addition).

For the fixed realization i, we can count the number of disjoint regions of $\chi^+_{w,i}$ ($\chi^-_{w,i}$) above the threshold t (below $-t$) as illustrated by the red (green) numbers in 1**a**. The Euler characteristic for i is then simply the sum of these counts.

If we repeat this permutation scheme many (N) times, we can estimate the expectation across realizations as indicated by the formula at the bottom of 1**a**.

An analytical approximation of the expected Euler characteristic. For many scales w (when w_L and w_R are large), we can accurately approximate $\bar{\chi}_w(t)$ analytically as illustrated in Supplementary Fig. 1**b**. We present our copy number data as a $S \times G$ matrix of copy number log ratios $[c_{s,g}]$, where S is the number of samples and G the number of probe measurements on the genome. Each row in the matrix represents a single sample profile c_s, where probes are sorted on the genome. Each sample profile can be convolved with the kernel to produce $t_{w,s} = k_w * c_s$.

First we compute the histogram for each sample's convolved profile ($t_{w,s}$). Note that these histograms remain unchanged for cyclically shifted profiles. These histograms have mean zero (the kernel integrates to zero) and are somewhat symmetric (positive breaks are as likely to occur as negative breaks in every sample). Due to independence between samples in the null, we can compute the histogram on the aggregate convolved profile by convolving all the sample histograms. It is then a consequence of the central limit theorem that allows us to approximate the convolved aggregate profile as a multivariate Gaussian random process with zero mean. Furthermore, this process will be stationary due to the cyclic shift hypothesis (all probes behave in the same way everywhere, since we randomly offset them for each realization). For a stationary zero-mean multivariate Gaussian process there are only two parameters that need to be estimated: The variance σ_w^2 and auto-correlation r_w. The auto-correlation is a function of Δg with $r_w(\Delta g)$ equal to the Pearson correlation between probe measurements separated by Δg probes. It turns out that, in order to relate $\bar{\chi}_w$ to a threshold t, we only need to compute $\rho_w = r_w(1)$, i.e. the Pearson correlation between adjacent probe measurements. We explicitly compute the variance σ_w^2 and ρ_w as follows:

$$\sigma_w^2 = \sum_{s=1}^{S} \sigma_{w,s}^2, \qquad \sigma_{w,s}^2 = \frac{1}{G}\sum_{g=1}^{G} t_{w,s}^2(g)$$

$$\rho_w = \frac{1}{\sigma_w^2}\sum_{s=1}^{S} \rho_{w,s}, \qquad \rho_{w,s} = \frac{1}{G}\sum_{g=1}^{G} t_{w,s}(g)\, t_{w,s}\big((g+1)/G\mathbb{Z}\big) \tag{2}$$

We can then accurately relate the expected Euler characteristic to a positive threshold t for a

stationary Gaussian process as follows:

$$\begin{aligned}\bar{\chi}_w(t) &= \frac{(G-1)V_w}{\pi}\exp\big(-\frac{1}{2}(t/\sigma_w)^2\big), \qquad \text{where} \\ V_w &= \arccos(\rho_w) \end{aligned} \tag{3}$$

RUBIC segmentation based on both the permutation and analytically derived expected Euler characteristic. In RUBIC segmentation, we need to compute $\bar{\chi}_w$ for many different scales. To do so purely based on the permutation scheme is computationally prohibitive and it is desirable to use the analytical estimate instead.

The Gaussian assumption does hold for the majority of kernel choices w. However, when w_L and w_R are small, the approximation becomes inaccurate. For example, suppose we choose $w = (1, 1)$. In this case, $t_{w,s}$ will be zero everywhere except at locations where copy number breaks occur. Due to the sparsity of $t_{w,s}$, the Gaussian assumption fails and the analytical prediction will be liberal.

Due to these considerations we need to perform segmentation using a hybrid between these estimates. The methodology is simple: We cluster segments based purely on the analytical model at first. After segmenting with this methodology, there will only be a small number of jump discontinuities in the segmented profile. All of these jump discontinuities will be significant according to the analytical estimate. It is only at this point where we recompute significance values (on the small set of jump discontinuities remaining) based on the permutation scheme. After we did so, we continue segmenting with the analytical estimates. We iteratively continue until all jump discontinuities are significant based on the permutation estimate.

With this procedure, we are always ensured that the expected number local maximum segments (that we end up calling) in the aggregate segmented profile is below or equal to $E/2$, where E is the global threshold used to stop clustering. This is true no matter what analytical approximation we use for $\bar{\chi}_w$. If this analytical approximation is conservative, we end up merging segments with jump discontinuities that should have been called significant and therefore the number of local maximum segments will be lower than $E/2$. On the other hand, if the analytical approximation is liberal, we do not lose power or incur more false positives. Instead, we end up testing many jump discontinuities with the permutation scheme. The analytical approximation we use tend to be liberal for small kernel sizes (and we should not expect to lose power because of it). Nevertheless, by the time we stop merging segments, the majority of jump discontinuities remaining define larger kernels, where the analytical approximations hold well.

Due to the overall accuracy of our analytical estimate, we rarely need to iterate these steps. Usually after segmenting the aggregate profile with the analytical approximations, the permutation scheme also calls the jump discontinuities significant and we stop.

Iteratively updating the null-model

Generally, we consider a copy number aberration to be a driver if it provides a selective advantage in tumor initiation and progression. Driver aberrations are likely to effect (and therefore overlap) with oncogenes and tumor suppressor genes (driver genes). Passenger aberrations on the other hand are believed to provide no significant selective advantage and occur due to genomic instability. It is not necessarily true that all aberrations that overlap with driver genes are driver aberrations. For example, it could happen that an aberration amplifies an inactive allele of an oncogene. Nevertheless, if we can prove that neutral-to-gain (or gain-to-neutral) breaks recur significantly across samples, then we have good reason to believe that at least a subset of them belong to driver aberrations.

In Supplementary Fig. 2 we show an example of 20 DNA copy number samples (only the gains). In the top panel we show all the driver aberrations in red, whereas passenger aberrations are shown in grey. Note that we generally don't have prior knowledge on which aberrations are drivers and we show it here only for illustrative purposes. The important point here is that not all driver aberrations (or breaks) will necessarily recur significantly. Therefore it is not possible to fully discriminate drivers from passengers in the data.

The cyclic shift null-model describes the behavior of passenger aberrations and is based on the assumption that they occur randomly on the genome. Unfortunately, this scheme will be conservative, since the overall break density will be higher in the cyclic null model than the true passenger break density. Although the break locations are random in the null, a large portion of the breaks that are scatter across the genome originate from drivers that are concentrated in fixed genomic loci in the data (see the lower panel in Supplementary Fig. 2). Therefore, the cyclic shift null-model will over estimate the background break density. Nevertheless, we can significantly reduce this bias by iteratively detecting recurrent break points with RUBIC and then remove the concentrated breaks from our null model. Notably, this will also automatically remove the apparent breaks between adjacent chromosome boundaries.

At each iteration we perform RUBIC segmentation on the aggregate profile with family wise error (FWER) control (it is not particularly natural to do FDR control for updating the null). Family wise error control is straight forward and achieved by setting the clustering threshold at $E =$ FWER without applying the Benjamini-Hochberg procedure. We set the family wise error rate equal to the FDR level. We segment each sample using the break locations detected by RUBIC (with segment amplitudes equal to the mean in that particular sample's copy number ratio). From each sample, we subtract this segmented profile and update the null accordingly (i.e. cyclically permute based on the new sample profiles). As a result, all frequently recurrent breaks are canceled. Note that the 'cancellation' of breaks will not necessarily align perfectly with the correct driver breaks. This leads to a slight power loss (slightly more conservative than need be) since we are technically introducing new breaks locally (and therefore increase the break density in the null). Nevertheless they will occur in close proximity (and be of opposite sign) to the true driver breaks

and greatly improves statistical power (especially for larger kernels).

At each iteration, we re-segment the aggregate of the original dataset (not the profiles with the cancelation breaks) based on the updated null-model. We continue iterating until no new breaks can be detected at the specified FWER level.

We use the resulting null-model (after convergence) to finally segment the aggregate with FDR control.

Evolutionary model for simulating copy number profiles

Simulating passenger aberrations. When we simulate tumor instability in our simulation model, we randomly add copy number aberrations that were extracted from the TCGA breast cancer datasets. Each copy number sample in the Level 3 TCGA data is represented as a list of segments with start and end positions and the average copy number log ratio of each segment. A copy number aberration does not typically correspond to these segments directly. For example, suppose there are only two aberrations in chromosome 1q, one focal amplification and one broad gain of the whole chromosome arm. We want to extract these two events separately, but in the level 3 data at our disposal there will be three segments corresponding to 1q. To extract aberrations we follow a simple strategy. In each sample and in each chromosome, we extract the segment with the highest (positive or negative) copy number log ratio. We then merge adjacent copy number segments that border the chosen segment into one large segment. We take care to associate the merged segment with a log ratio that is the weighted average of the two segments contributing (the weights depend on the sizes of the segments). We iteratively extract segments in this fashion until no more segments remain.

This procedure is performed for each sample and each chromosome in our TCGA breast cancer dataset. Since we use these segments to simulate passenger aberrations that are located at random positions and samples, we need not record their locations nor which samples they are from, but only their genomic widths and log ratio values. We do add a flag indicating whether the segment is as wide as the chromosome, i.e. was the last segment extracted from a complete chromosome. Therefore we end up with a large aberration list with three fields: the width, log ratio and chromosome wide flag.

When we add a passenger aberration to an existing profile, we select a random chromosome (the probability of each chromosome is weighted by its length) and a random aberration from the list described above. If the chosen segment is flagged as chromosome wide, we change its size to the chromosome width and center it to cover the whole chromosome. Otherwise, we center the aberration at a random position in the chromosome and clip the segment at chromosome boundaries if necessary. Finally, we add the segment to the existing profile.

Computing the proliferation score. We defined a list of oncogenes and tumor suppressors d_i ($1 \leq i \leq D$). For each we assigned a proliferation coefficient α_i where positive (negative) values are associated with oncogenes (tumor suppressors). From this we can compute a proliferation score P for any fixed copy number profile by assuming that cell proliferation is linearly related to the average copy number dosage (f_i) (of gene d_i) and α_i:

$$P = \sum_{i \in \{1,\ldots,D\}} \alpha_i f_i \tag{4}$$

The dosage f_i of gene d_i is based on the average copy number log ratio $\bar{c_i}$ of the gene and is a measure of the fold change relative to a normal reference:

$$f_i = \text{sign}(\bar{c_i})(A^{|\bar{c_i}|} - 1), \tag{5}$$

where $A = 2$ is the log base.

Parameter choices for RUBIC, GISTIC2 and RAIG

We used a fixed set of parameters for each algorithm on all simulation and TCGA datasets.

Preprocessing parameters common to all algorithms. All three algorithms split DNA copy number gains and losses. All three algorithms accomplish this by defining a positive and negative log ratio threshold. To obtain copy number profiles containing only gains, we only consider segments with copy number values above the positive threshold and set all remaining segments to the copy number neutral (log ratio equal to zero) state. Deletion only profiles are obtained in a symmetric fashion with the negative log ratio threshold. For all three algorithms we used thresholds at -0.1 and 0.1. This is also the default for both GISTIC2 and RAIG.

For all three algorithms, we also clipped single sample log ratio values at 1.5 (default in GISTIC2). This means that if a segment in a single sample has a log ratio above 1.5 (below -1.5), we set it equal to 1.5 (-1.5).

GISTIC2 parameters. The GISTIC2 analysis was performed (independently of us) in the http://firebrowse.org/ (downloaded on 31 March 2015) pipeline and on the exact same TCGA datasets. In this pipeline, all parameters were the same for all three datasets and are specified as follows:

- Amplification Threshold = 0.1
- Deletion Threshold = 0.1
- Cap Values = 1.5
- Broad Length Cutoff = 0.7
- Remove X-Chromosome = 0
- Confidence Level = 0.99

- Join Segment Size = 4
- Arm Level Peel Off = 1
- Maximum Sample Segments = 2000
- Gene GISTIC = 1
- q-value threshold = 0.25

RAIG parameters. RAIG was performed on a subset of the TCGA Breast cancer and Glioblastoma datasets that we considered in [29]. For these two datasets, RAIG used the same parameter settings than we did with two exceptions. First, RAIG has a flag specifying whether the analysis should be performed on the gene level. For the Glioblastoma set, this flag was set to 'off' (in contrast to other data sets) in [29]. We set this flag to 'on', since we are interested in discovering genes. Second, in [29] the q-value threshold (that corresponds to the false discovery rate) was set at 0.5 which we set at 0.25. Otherwise all parameters are the same:

- δ lower bound = 0.05
- Maximum size of a block (percentage of genome size) = 0.1
- Gene level = on
- Target region selection t = 1
- q-value threshold = 0.25

Pre-processing of Next Generation Sequencing (NGS) data sets

Low coverage Whole Genome Sequencing data. We gathered breast cancer tumor samples for 90 patients that are independent of the TCGA collection (See [30] and references therein). The segmented data is available on figshare: figshare.com/s/f82d18da993411e5961706ec4bbcf141 with the following associated DOI: http://dx.doi.org/10.6084/m9.figshare.1615908. This collection of samples is highly enriched for BRCA1/2-like, mutated and methylated samples. 36 (40%) of the samples are triple-negative and at least 31 (34%) are believed to be BRCA1 deficient (either BRCA1-like, mutated or methylated). 14 (16%) additional samples are also predicted to be BRCA2-like. These sample appear to have highly unstable genomes and the estimated number of breakpoints per sample is 146 on average. All copy number profiling and processing was performed as described in previous publications (See [30] and references therein). The un-segmented data (i.e. raw pre-processed data, according to established methods per platform) were used as input in the analysis. The profiles were segmented with *cghseg*[31]. As no matched normal samples were available, the germline CNVs were labeled manually and removed from the data. Segmented profiles for 90 tumors were employed as input to both RUBIC and GISTIC2.

Whole Exome Sequencing data. In total there were 754 primary TCGA BRCA tumors with matched normal (peripheral blood) for which WES sequencing data was available for both the tumor and normal (available on https://cghub.ucsc.edu/). We downloaded 792 bam files for a randomly selected set of samples (396 tumors + 396 matched normals). Exome coverage is prone to

batch effects, introduced, for example, by capture kits, amplification steps and sequencing platforms. We performed hierarchical clustering on the Euclidean distance between all samples based on the on-target (exon) read counts to determine batches. Thirteen samples were removed from further analysis as they could not be assigned to any cluster. For seven of these we know that whole genome amplification was performed prior to sequencing, which may explain the observed coverage bias. Per cluster, normal samples were combined into a reference pool and both tumor and normal samples were compared to this combined reference using *CNVkit*[32] to obtain segmented copy number profiles. The normal sample profiles were used to identify germline CNVs. Segments in each tumor sample with a Jaccard similarity coefficient of more that 20% with a germline CNV (i.e. overlaps with a germline CNV) were removed from the data set. Segmented profiles for 383 tumors were employed as input to both RUBIC and GISTIC2.

Construction of the *bona fide* breast cancer gene list

We compiled a list of 52 breast cancer oncogenes and 12 breast cancer tumor suppressor genes from the literature. A subset of the oncogenes in this list were only recently validated[5] and a large fraction were proposed by Santarius and colleagues based on the criteria as described in their publication [33]. The full list of breast cancer genes is given in Supplementary Table 1 with the associated literature references.

Fragile site analysis

First we employed a published list[34] of fragile sites and combined that with an unpublished list of fragile sites obtained from the Sanger Institute to construct a list of 127 rare and common fragile sites (Supplementary Data 12). To reject the null hypothesis that fragile sites are not enriched for RUBIC recurrent regions, we performed a permutation-based enrichment test. Specifically, as statistic, we employed the number of recurrent regions called by RUBIC that overlapped with at least one fragile site. We tested whether this statistic is significant based on the null distribution. To generate the null distribution, we collected 10000 values of the statistic under the null, by performing random cyclic permutation of the recurrent regions called by RUBIC and computing the overlap with at least one fragile site. From the statistic on the unpermuted data and the null distribution we computed a single-tail p-value for the observed number of overlaps in the data. Amplifications and deletions were considered independently. All datasets were also considered independently.

Lists of detected RUBIC and GISTIC2 regions with gene priority scores

We constructed Excel files for each data set considered. Amplifications and deletions are also represented in separate files. Each row in an Excel file represents a genomic region detected by either RUBIC or GISTIC2. The regions are sorted based on genomic location so that overlapping

RUBIC and GISTIC2 regions are next to each other. Each row has eight fields.

- Method. The algorithm with which the region was discovered. This is either RUBIC or GISTIC2.
- Negative log q-value. This is the negative log of the region's significance. Large positive values represent highly significant regions.
- Chr. The chromosome in which the region was detected.
- Start. The left genomic boundary of the detected region.
- End. The right genomic boundary of the detected region.
- *Bona fide* (BRCA). The list of *bona fide* breast cancer oncogenes or tumor suppressors that overlap with the region for amplifications and deletions respectively.
- All genes. A list of all the genes that overlap the region.
- Citation score. This is a measure of how frequently each overlapping gene is mentioned in cancer specific research publications with Pubmed IDs.

In order to compute the citation score, we counted the number of cancer research publications with Pubmed IDs that explicitly mention each gene. We ranked genes based on this count. The citation score is then obtained by computing the fraction of genes that score a lower citation count[35].

We created a set of supplemental Supplementary Data Excel files containing the information of all the aberrations for both RUBIC and GISTIC2 in the same file to ease comparison. Each of these files contains a header describing the information contained in the file. Below we also list the Supplementary Data file name, followed by the information contained in the file:

1. Supplementary Data 2:
    - Aberration type: Recurrent Amplifications
    - Cancer type: Breast Cancer (BRCA)
    - Profile type: SNP6 profiles
    - Algorithms: RUBIC and GISTIC2
2. Supplementary Data 3
    - Aberration type: Recurrent Deletions
    - Cancer type: Breast Cancer (BRCA)
    - Profile type: SNP6 profiles
    - Algorithms: RUBIC and GISTIC2
3. Supplementary Data 4
    - Aberration type: Recurrent Amplifications
    - Cancer type: Glioblastoma Multiforme (GBM)
    - Profile type: SNP6 profiles

- Algorithms: RUBIC and GISTIC2

4. Supplementary Data 5
    - Aberration type: Recurrent Deletions
    - Cancer type: Glioblastoma Multiforme (GBM)
    - Profile type: SNP6 profiles
    - Algorithms: RUBIC and GISTIC2
5. Supplementary Data 6
    - Aberration type: Recurrent Amplifications
    - Cancer type: Colon Adenocarcinoma (COAD)
    - Profile type: SNP6 profiles
    - Algorithms: RUBIC and GISTIC2
6. Supplementary Data 7
    - Aberration type: Recurrent Deletions
    - Cancer type: Colon Adenocarcinoma (COAD)
    - Profile type: SNP6 profiles
    - Algorithms: RUBIC and GISTIC2
7. Supplementary Data 8
    - Aberration type: Recurrent Amplifications
    - Cancer type: Breast Cancer (BRCA)
    - Profile type: Whole Exome Sequencing (WES)
    - Algorithms: RUBIC and GISTIC2
8. Supplementary Data 9
    - Aberration type: Recurrent Deletions
    - Cancer type: Breast Cancer (BRCA)
    - Profile type: Whole Exome Sequencing (WES)
    - Algorithms: RUBIC and GISTIC2
9. Supplementary Data 10
    - Aberration type: Recurrent Amplifications
    - Cancer type: Breast Cancer (BRCA)
    - Profile type: low coverage Whole Genome Sequencing (lcWGS)
    - Algorithms: RUBIC and GISTIC2
10. Supplementary Data 11
    - Aberration type: Recurrent Deletions
    - Cancer type: Breast Cancer (BRCA)
    - Profile type: low coverage Whole Genome Sequencing (lcWGS)
    - Algorithms: RUBIC and GISTIC2

Derivation of the analytical approximation of the expected Euler characteristic.

We will now derive an accurate analytical expression relating the expected Euler characteristic to a fixed non-negative threshold t for a discrete Gaussian random process with constant variance σ^2, zero mean and non-stationary correlation function $r(g)$. In our application we have a discrete stationary Gaussian random process defined at a finite number of probes $\big(T_i : i \in \{0, 1, ..., G\}\big)$, but the theory applies to non-stationary processes too. Much work has been done on estimating the expected Euler characteristic in stationary and discrete processes [36–38]. These estimates are typically not very accurate when adjacent probes are weakly (or negatively) correlated. On the other had, for smooth stationary Gaussian processes, there exist exact expressions for the expected Euler characteristics [39]. Our strategy will be to interpolate discrete processes with a smooth (in the sense that it is continuously differentiable up to any order) Gaussian random process $H(g)$ (Supplementary Fig. 3). From the smoothed process we can derive an extremely simple and exact expression for the expected Euler characteristic.

Without loss of generality, we will assume that the discrete process has a variance equal to one for all probes. For simplicity, we assume that the Euler characteristic is equal to the sum of the up-crossings (the red circles in Supplementary Fig. 3) and down-crossings (the green circle in Supplementary Fig. 3) at the thresholds t and $-t$ respectively. We refer to these up-crossings and down-crossings collectively as the level-crossings of t. Strictly, if $|t_0| > t$, where t_0 is the left most probe measurement, the Euler characteristic will be equal to the number of crossing points plus one. Although it is easy to correct for this boundary effect, it is usually negligible and we will not consider it any further in this section.

Traditionally, the expected Euler characteristic is only computed for up-crossing above a positive threshold. In our application, we also count the down-crossings. Since a Gaussian random process is symmetric with respect to the zero line, the expected number of level crossings will be double the number of up-crossings.

The reason we prefer to work with a smooth random process is due to the following theorem that can be found in the supplementary data in [40]:

Theorem 1. *Consider a non-negative threshold* t*, a closed interval* $R = [g_L, g_R]$ *and a suitably regular (non-stationary) random Gaussian process* H *with* $\forall g \in \mathbb{R}\ H(g) \sim N(0, 1)$*. The expected number of level crossings for a threshold* t *in* R *is equal to:*

$$\bar{\chi}(t) \quad = \quad \frac{e^{-t^2/2}}{\pi} \int_R \sqrt{Var[\frac{d}{dg} H(g)]} \tag{6}$$

For the definition of a suitably regular process, see the supplementary data in [40].

To smooth the discrete process, we convolve it with a particular bump function f satisfying the following properties:

- It is smooth (continuously differentiable up to any order).
- $\{g : f(g) = 0\} = (-\infty, 1] \cup [1, \infty)$
- $\{g : f(g) > 0\} = (-1, 1)$
- $f(0) = 1$
- $\forall 0 \leq g \leq 1$, f(g) + f(g - 1) = 1

The exact choice of this function is not important, however a good example is illustrated in Supplementary Fig. 4 and is defined as follows:

$$\begin{aligned}
f(g) &= \Gamma(g)/\Gamma(0), \quad \text{where} \\
\Gamma(g) &= \int_{-1}^{g} \beta(2u+1) - \beta(2u-1) du \\
\beta(g) &= \gamma(1-g)\gamma(1+g) \\
\gamma(g) &= \begin{cases} e^{-1/g} & g > 0 \\ 0 & g \leq 0 \end{cases}
\end{aligned} \tag{7}$$

We convolve a realization of the discrete process $\big(t_i : i \in \{0, 1, ..., G-1\}\big)$ with f to produce the smoothed profile:

$$s(g) \;=\; f(g - \lfloor g \rfloor) t_{\lfloor g \rfloor} + \big(1 - f(g - \lfloor g \rfloor)\big) t_{\lfloor g \rfloor + 1} \tag{8}$$

Note that at any particular position g, s only depends on the two adjacent random variables $t_{\lfloor g \rfloor}$ and $t_{\lfloor g \rfloor+1}$. The covariance matrix of two variables t_i and t_{i+1} is presented as follows:

$$\Sigma_i \;=\; \begin{bmatrix} 1 & \rho_i \\ \rho_i & 1 \end{bmatrix}, \tag{9}$$

where $\rho_i = \text{Cov}(t_i, t_{i+1})$. Using this, we can easily compute the variance of S at any position g:

$$\begin{aligned}
\text{Var}[S(g)] &= [f_g, 1 - f_g] \Sigma_{\lfloor g \rfloor} [f_g, 1 - f_g]^T, \quad \text{where} \\
f_g &= f(g - \lfloor g \rfloor)
\end{aligned} \tag{10}$$

Generally, Var$[S(g)]$ is equal to one at integer values, but strictly smaller at intermediate values. Therefore, our final step in smoothing the discrete process is to z-normalize. The final

smoothed interpolation function of a realization of the discrete random process is therefore defined as follows:

$$h(g) \;=\; \frac{s(g)}{\sqrt{\mathrm{Var}[S(g)]}} \tag{11}$$

In Supplementary Fig. 3 we show an example realization of a discrete process (with blue dots) and the resulting smoothed function $h(g)$ in red.

Theorem 2. *Consider a non-negative threshold t, a non-stationary discrete random Gaussian process $(T_i : i \in \{0, 1, ..., G-1\})$ with $\forall i \in \{0, 1, ..., G-1\}$ $T_i \sim N(0, 1)$. The Expected number of level crossings of the interpolation process $H(g)$ in the region $[0, G-1]$ is equal to:*

$$\bar{\chi}(t) \;=\; \frac{e^{-t^2/2}}{\pi} \sum_{i=0}^{G-2} \arccos(\rho_i) \tag{12}$$

Proof. Since f is smooth and h is an algebraic expression of f, h is itself smooth and $\forall g \in [0, G-1], \mathrm{Var}[H(g)] = 1$. As a consequence, all the conditions in Theorem 1 are satisfied. The only part that needs a proof is:

$$\int_0^1 \sqrt{\mathrm{Var}\big[\frac{d}{dg} H(g)\big]}\, dg \;=\; \arccos(\rho_0), \tag{13}$$

For any $g \in [\lfloor g \rfloor, \lfloor g \rfloor + 1]$, only the variables $T_{\lfloor g \rfloor}$ and $T_{\lfloor g \rfloor + 1}$ are involved in an analogous manner as T_0 and T_1 are involved for $g \in [0, 1]$. From this observation and Equation 13, Theorem 2 immediately follows.

In all subsequent steps, we only consider $g \in [0, 1]$. First we can simplify Equation 10:

$$\begin{aligned} C = \mathrm{Var}[S(g)] \;&=\; [f, 1-f]\Sigma_0[f, 1-f]^T \\ &=\; 2\alpha f^2 - 2\alpha f + 1, \end{aligned} \tag{14}$$

where $\alpha = 1 - \rho_0$. Next we rewrite Equation 11 as follows:

$$h = \frac{f t_0 + (1-f) t_1}{\sqrt{C}} = \frac{f}{\sqrt{C}}(t_0 - t_1) + \frac{1}{\sqrt{C}} t_1 \tag{15}$$

Taking the derivative yields

$$h' = (p + qf)(t_0 - t_1) + q t_1, \tag{16}$$

where

$$p = f' C^{-1/2}, \;\; q = -\frac{1}{2} C^{-3/2} C' \tag{17}$$

It is easy to show that:

$$\mathrm{Cov}(t_0 - t_1, t_1) = \begin{bmatrix} 2\alpha & -\alpha \\ -\alpha & 1 \end{bmatrix}, \tag{18}$$

As a consequence,

$$\mathrm{Var}[H'] = [p + qf, q] \begin{bmatrix} 2\alpha & -\alpha \\ -\alpha & 1 \end{bmatrix} [p + qf, q]^T, \tag{19}$$

After a long, but straightforward, derivation we obtain the following:

$$\begin{aligned} \sqrt{\mathrm{Var}[H']} &= \frac{r'}{r^2 + 1} \\ r &= \sqrt{\frac{1 - \rho_0}{1 + \rho_0}}(2f - 1) \end{aligned} \tag{20}$$

If we set $r_0 = -\sqrt{\frac{1-\rho_0}{1+\rho_0}}$ and $r_1 = \sqrt{\frac{1-\rho_0}{1+\rho_0}}$,

$$\begin{aligned} \int_0^1 \sqrt{\mathrm{Var}[H']} dg &= \int_{r_0}^{r_1} \frac{r'}{r^2 + 1} dr \\ &= \arctan(r_1) - \arctan(r_0) \\ &= \arccos \rho_0 \end{aligned} \tag{21}$$

□

The clustering threshold E controls the expected number of false positives

Overview. If we perform hierarchical clustering on a realization of the null model and stop merging segments when all similarity measures ($\bar{\chi}$) between adjacent segments are below a global threshold E, then there will be a certain number of local maximum segments. The purpose of this section is to show that the expected number of local maximum segments resulting across realizations will be less than or equal to $E/2$. In this section it is not necessary to assume that the null process (describing the aggregate) is Gaussian. However, there are a number of properties that are required for the null model. Unfortunately we need to introduce some details and therefore we introduce these properties gradually. All these properties hold in the cyclic permutation scheme that we use. We start with the first two properties

- Property 1: The distribution of the aggregate of a probe (across realizations) is independent of the probe index, i.e. all probes have the same distribution.
- Property 2: The aggregate process is stationary, i.e. the covariance between probe measurements in the aggregate depends only on the index distance between them and not the actual probe indices

The Euler characteristic at a fixed scale $w = (w_L, w_R)$ and fixed positive threshold t in the null model. For a fixed scale we convolve the aggregate profile of a fixed realization of the null with a kernel k_w which results in values $(t_g : 0 \leq g \leq G-1)$, where G is the number of probes. Strictly, $t_g = t_w(g)$ is a function of the scale $w = (w_L, w_R)$ and we drop this for convenience. The corresponding null process $(T_g : 0 \leq g \leq G-1)$ is stationary and have mean zero everywhere, since the kernel integrates to zero. Next, we introduce the third property of the null:

- Property 3: The distribution of each T_g is symmetric with respect to zero.

This assumption holds, since our null model describes the behavior of passenger aberrations on the genome. Positive (neutral-gain) breaks are as likely to occur as negative (gain-neutral) breaks in each sample and their locations are also random on the genome.

For a fixed threshold t we can compute the Euler characteristic by counting crossings as indicated in Supplementary Fig. 5. There are two possible ways in which $(t_g : 0 \leq g \leq G-1)$ can cross the positive threshold t:

- Up-crossings: probe g is considered an up-crossing when $t_g \geq t$ and $t_{g-1} < t$. In Supplementary Fig. 5 we mark these events with green circles.
- Down-crossings: $t_g \leq t$ and $t_{g-1} > t$. These are the blue circles in Supplementary Fig. 5.

By convention we never count a crossing at probe index 0, since there is no probe to the left for comparison.

We count the number of up-crossings and down-crossings and denote it with $\chi_w^{\uparrow}(t)$ and $\chi_w^{\downarrow}(t)$ respectively. Similarly we can count the number of crossings at threshold $-t$ and denote it with $\chi_w^{\uparrow}(-t)$ (red circles) and $\chi_w^{\downarrow}(-t)$ (orange circles). In this notation, the expected Euler characteristic that we use as a similarity measure is equal to $\bar{\chi}_w(|t|) = E\left[\chi_w^{\uparrow}(t) + \chi_w^{\downarrow}(-t)\right]$, where the expectation is across realizations. As a consequence of Property 3 and the fact that expectation is a linear operator, we can see that:

$$E\left[\chi_w^{\uparrow}(t)\right] = E\left[\chi_w^{\downarrow}(t)\right] = E\left[\chi_w^{\uparrow}(-t)\right] = E\left[\chi_w^{\downarrow}(-t)\right] = \frac{1}{2}\bar{\chi}_w(|t|) \tag{22}$$

Counting up-crossing at t across the whole genome is equivalent to summing up-crossings in sub-intervals as follows:

$$\chi_w^{\uparrow}(t) = \chi_{w,\{0,\dots,g_0\}}^{\uparrow}(t) + \chi_{w,\{g_0,\dots,g_1\}}^{\uparrow}(t) + \dots + \chi_{w,\{g_n,\dots,G-1\}}^{\uparrow}(t) \tag{23}$$

Note that we include g_0 in both the intervals $\{0, \dots, g_0\}$ and $\{g_0, \dots, g_1\}$. This is because we never count the crossing in the left most probe of an interval (to stay in accordance with our definition).

From this observation, properties 1, 2 and, again, linearity of expectation, we see that

$$\bar{\chi}_{w,\{g_1,\dots,g_2\}}^{\uparrow}(t) = E\left[\chi_{w,\{g_1,\dots,g_2\}}^{\uparrow}(t)\right] = \frac{g_2 - g_1}{2(G-1)}\bar{\chi}_w(|t|) \tag{24}$$

We can define $\bar{\chi}_{w,\{g_1,\dots,g_2\}}^{\downarrow}(t)$, $\bar{\chi}_{w,\{g_1,\dots,g_2\}}^{\downarrow}(-t)$ and $\bar{\chi}_{w,\{g_1,\dots,g_2\}}^{\uparrow}(-t)$ in a similar fashion and note that these expectations are all equal.

It is convenient to work with a density measure on the expected Euler characteristic. This density is exactly equal to the expected number of crossings at one probe (or the expected Euler characteristic at one probe). A single probe cannot be more than one type of crossing and we are therefore computing the expectation of a Bernoulli trail which is therefore also exactly equal to the probability of a probe to be a crossing. Due to the previous equation and Equation 22 we can compute these densities as follows (setting $g_2 = g_1 + 1$):

$$\Delta\bar{\chi}_w(|t|) = \frac{1}{G-1}\bar{\chi}_w(|t|)$$
$$\Delta\bar{\chi}_w^{\uparrow}(t) = \Delta\bar{\chi}_w^{\downarrow}(t) = \Delta\bar{\chi}_w^{\uparrow}(-t) = \Delta\bar{\chi}_w^{\downarrow}(-t) = \frac{1}{2}\Delta\bar{\chi}_w(|t|) \tag{25}$$

As an application, say we wish to compute the expected number of up-crossings at the negative threshold -t over P probes. Then we simply compute $\frac{P-1}{2}\Delta\bar{\chi}_w(|t|)$.

It is also convenient to transform the vector $(t_g : 0 \leq g \leq G-1)$ into a new vector $(\tau_g : 0 \leq g \leq G-1)$, where each τ_g represents a significance measure in terms of the expected Euler characteristic. For each t_g we can compute $\bar{\chi}_w(|t_g|)$, which is always positive and is uninformative with respect to the sign of t_g. Furthermore, a large value t_g will correspond to a low value for $\bar{\chi}_w(|t_g|)$. For the sake of convenience, we would like τ_g to be an increasing function of t_g, i.e. large values in τ_g represent highly significant. With these considerations in mind, we define τ_g as follows:

$$\tau_g = \tau_w(g) = -\text{sign}(t_g)\log(\frac{\bar{\chi}_w(|t_g|)}{G-1}) \tag{26}$$

We should note that for any scale w and value t_g, $\bar{\chi}_w(|t_g|) \leq G-1$, since the number of crossing points can never exceed the number of probes minus one (probe zero is never a crossing). Therefore the log will always be non-positive. τ_g will always have the same sign and be an increasing function of t_g. By convention, we also set sign$(0) = 0$.

Counting crossing in $(t_g : 0 \leq g \leq G-1)$ with respect to a positive threshold t is equivalent to counting crossings in $(\tau_g : 0 \leq g \leq G-1)$ with respect to the threshold $T = -\log(\frac{\bar{\chi}_w(t)}{G-1})$. We can also express the expected Euler characteristic density in Equation 25 in terms of the threshold T:

$$\begin{aligned} \Delta\bar{\chi}(T) &= \Delta\bar{\chi}_w(|t|) = e^{-T} \\ \Delta\bar{\chi}^{\uparrow}(T) = \Delta\bar{\chi}^{\downarrow}(T) = \Delta\bar{\chi}^{\uparrow}(-T) = \Delta\bar{\chi}^{\downarrow}(-T) &= \frac{1}{2}\Delta\bar{\chi}(T) \end{aligned} \tag{27}$$

This form is extremely convenient, since $\Delta\bar{\chi}$ only depends on T and not the scale w.

As before, we can also count the number of crossing in a restricted interval and denote them by $\chi^{\uparrow}_{\{g_1,\ldots g_2\}}(T)$, etc. We can compute the expected number of up-crossings at T in a restricted interval $\{g_1, \ldots g_2\}$ as follows: $\bar{\chi}^{\uparrow}_{\{g_1,\ldots g_2\}}(T) = \frac{g_2-g_1}{2}\Delta\bar{\chi}(T)$.

Counting local extrema in the segmented aggregate. In agglomerative clustering, we continue merging segments until all similarity measures between adjacent segments are smaller than a global threshold E. A hypothetical realization of the null after segmenting the aggregate is illustrated in Supplementary Fig. 6a. The blue dots represent the aggregate of each probe, while the piecewise constant red graph represent the aggregate profile after clustering. Each segment has a height equal to the mean aggregate. By convention, we define a local extremum as a segment that is supported by two jump discontinuities of opposite sign. As a consequence, we do not count segments on the boundaries as local extrema. In Supplementary Fig. 6a, there are exactly three local extrema.

In Supplementary Fig. 6b we computed the difference between adjacent probes in the segmented profile. This results in a sparse function that is equal to zero everywhere except perhaps at the jump discontinuities. The height of a jump discontinuity at location g_n is exactly equal to

$t_{g_n} = t_{(\omega_n,\omega_{n+1})}(g_n)$. In Supplementary Fig. 6c, we map each t_{g_n} to its corresponding expected Euler characteristic $\bar{\chi}_{(\omega_n,\omega_{n+1})}(|t_{g_n}|)$. These are the similarity measures that we used for clustering and are all below the threshold E since this is the point at which we stopped merging. In Supplementary Fig. 6d we show the τ profile, where each t_{g_n} is converted to its respective τ_{g_n} value as explained earlier. Note that the threshold E in Supplementary Fig. 6d corresponds exactly to the threshold $T = -\log(\frac{E}{G-1})$.

The green ellipses in Supplementary Fig. 6d illustrates a different way in which we count local extrema. Counting local extrema is performed in exactly the same way we compute the Euler characteristic for a fixed scale, except that we:

- use the τ profile in Supplementary Fig. 6d,
- use the threshold T, and
- ignore all the probes where there are no jump discontinuities, i.e. we collapse the profile with new indices corresponding to the jump discontinuities.

We represent this number with $\chi(T)$. Note that $\chi(T)$ does not depend on any of the scale parameters, since we are counting on the segmented profile and not just any particular scale. Also note that for all τ values at the jump discontinuities it follows that $|\tau| \geq T$ (again because this is where we stop clustering). The above definition of $\chi(T)$ is only valid in this case. In the next section we show how to compute $\chi(T)$ in cases where $|\tau| < T$.

At the end of clustering, $\chi(T)$ will always be equal to the number of local extrema in the segmented profile (which equals three in Supplementary Fig. 6a). Generally, the number of local maximum segments will be approximately equal to half of the number of local extrema. To be exact:

- $\chi(T)/2$ if $\chi(T)$ is even,
- $(\chi(T)+1)/2$ if $\chi(T)$ is odd and the left most jump discontinuity $t_{(\omega_0,\omega_1)}(g_0)$ has a positive sign (this is the case in Supplementary Fig. 6), and
- $(\chi(T)-1)/2$ if $\chi(T)$ is odd and the left most jump discontinuity has a negative sign.

When segmenting a realization of the null, the chance that the left most break is positive is exactly 50%. The expected number of local maximum segments across realizations will therefore be exactly half of $E[\chi(t)]$.

The goal of this section is therefore to show that $E[\chi(T)] \leq E$.

Computing $\chi(T)$ when some τ values are below T. We just showed how to compute $\chi(T)$ when all jump discontinuities $|\tau_{g_n}| \geq T$. However, this is only the case at the terminal stage of clustering. For intermediate stages, there will be discontinuities where $|\tau_{g_n}| < T$. In general, the count $\chi(T)$ is computed in a slightly different manner than for a fixed scale.

We count crossings on the vector $(\tau_{g_n} : 0 \leq n \leq S-1)$, where S is the number of breaks in the segmentation. We define a discontinuity at index n as a T up-crossing if $\tau_{g_n} \geq T$ and $\tau_{g_{n-1}} < T$. By convention, the first discontinuity at index 0 will never be an up-crossing. We denote the set of indices that correspond to T up-crossings with C^+. Similarly, we define a discontinuity at index n as a $-T$ down-crossings if $\tau_{g_n} \leq -T$ and $\tau_{g_{n-1}} > -T$. We denote the set of indices that correspond to $-T$ down-crossings with C^-. Note that C^+ and C^- are disjoint.

We now introduce new types of crossings that we collectively refer to as switch crossings. We define a discontinuity at index n as a T switch-crossing if:

$$n \in C^+ \quad \text{and} \quad \max\{i \in C^- : i < n\} \geq \max\{i \in C^+ : i < n\} \tag{28}$$

Similarly, n is a $-T$ switch crossing if:

$$n \in C^- \quad \text{and} \quad \max\{i \in C^+ : i < n\} \geq \max\{i \in C^- : i < n\} \tag{29}$$

By convention, we define the max of an empty set to be equal to 0, i.e. $\max\{\} = 0$. We denote the sets of T and $-T$ switch-crossings with S^+ and S^-. Notably, $S^+ \subseteq C^+$, $S^- \subseteq C^-$. Also note that: $\min(C^+ \cup C^-) \in S^+ \cup S^-$, i.e. the left most crossing (at T or $-T$) is automatically a switch crossing. This is because the max will be 0 in both cases and the '$\geq$' statement becomes true.

The Euler characteristic is defined as the cardinality of $C^+ \cup C^-$. However, we define $\chi(T)$ as the cardinality of $S^+ \cup S^-$. Note that if $\forall n \in \{0, ..., S-1\}, |\tau_{g_n}| \geq T$, $\chi(T)$ will be equal to the Euler characteristic, otherwise it will be less than or equal.

We can also compute $\chi(T)$ when segmenting on a restricted number of probes $\{m, m+1, ..., n\}$ and we denote it with $\chi_{\{m,...,n\}}(T)$. We should be careful to note that we only segment on this restricted set of probes. Generally, the resulting segmentation will not be the same as when performed on $\{0, ..., G-1\}$, since there is no guarantee that jump discontinuities will result at m and n. Later on, however, we will only compute $\chi_{\{m,...,n\}}(T)$ when the global segmentation have jump discontinuities at m and n.

Merging adjacent segments in one iteration of clustering. Supplementary Fig. 7 illustrates the step in agglomerative clustering where two segments are merged with the lowest $|\tau|$ separating them at location g_n. The red graph in Supplementary Fig. 7a shows the segmented aggregate before merging segments of widths ω_n and ω_{n+1} at iteration k. The black graph shows the segmented aggregate after merging the segments at iteration $k+1$. In Supplementary Fig. 7b we show the lag-one difference profile of the segmented aggregate (similar to Supplementary Fig. 6b) before merging and in 7c we show the corresponding tau plot (similar to Supplementary Fig. 6d) before merging. In Supplementary Fig. 7b we show the thresholds in the diff graph that corresponds to $T = -\log(\frac{E}{G-1})$ in the τ profile. Note that these thresholds differ between discontinuities,

since they are all at different scales. Supplementary Fig. 7d and 7e are the same as 7b and 7c respectively, except that these relate to the segmented aggregate after merging segments (iteration $k+1$). The only differences between Supplementary Fig. 7c and 7e are:

- We remove the jump discontinuity corresponding to $\tau^k_{g_n} = \tau_{(\omega_n,\omega_{n+1})}(g_n)$
- We replace $\tau^k_{g_{n-1}} = \tau_{(\omega_{n-1},\omega_n)}(g_{n-1})$ with $\tau^{k+1}_{g_{n-1}} = \tau_{(\omega_{n-1},\omega_n+\omega_{n+1})}(g_{n-1})$
- We replace $\tau^k_{g_{n+1}} = \tau_{(\omega_n,\omega_{n+1})}(g_{n+1})$ with $\tau^{k+1}_{g_{n+1}} = \tau_{(\omega_n+\omega_{n+1},\omega_{n+2})}(g_{n+1})$

In this specific example, the new value $\tau^{k+1}_{g_{n+1}}$ is above the threshold T. If $\tau^{k+1}_{g_{n-1}}$ was also significant, we would stop merging at this point.

Jump discontinuities can become significant after merging. Case 1: bordering a significant discontinuity of the opposite sign. In Supplementary Fig. 7d we show $t_{(\omega_n+\omega_{n+1},\omega_{n+2})}(g)$ evaluated at every g (not just g_{n+1}). The important point to note here is that although $t_{(\omega_n+\omega_{n+1},\omega_{n+2})}(g_{n+1})$ is above the threshold $\bar{\chi}^{-1}_{(\omega_n+\omega_{n+1},\omega_{n+2})}(E)$, it is short lived with a crossing in close proximity. It is extremely likely that such a crossing will occur in the region $\{g_{n+1},...,g_{n+1}+\omega_n\}$. This is illustrated in Supplementary Fig. 7f. Here we show the kernel (in black) $k_{(\omega_n+\omega_{n+1},\omega_{n+2})}$ when shifted to $g_{n+1}+\omega_n$, i.e. $k_{(\omega_n+\omega_{n+1},\omega_{n+2})}(g_{n+2}+\omega_n-g)$. The value of $t_{(\omega_n+\omega_{n+1},\omega_{n+2})}(g_{n+2}+\omega_n)$ is evaluated by computing the average aggregate in the right lobe of the kernel and subtracting the average in the left lobe. These averages are shown by the dotted lines in Supplementary Fig. 7f.

As a consequence of this observation, we specify yet another property of the null model. Given a segmentation with jump discontinuities
$\{\tau_{(\omega_n,\omega_{n+1})}(g_n), \tau_{(\omega_{n+1},\omega_{n+2})}(g_{n+1}), \tau_{(\omega_{n+2},\omega_{n+3})}(g_{n+2})\}$ and an arbitrary positive thresholds T.

- Property 4: If $\tau_{(\omega_n,\omega_{n+1})}(g_n) < T$, $\tau_{(\omega_{n+1},\omega_{n+2})}(g_{n+1}) < T$ and $\tau_{(\omega_{n+2},\omega_{n+3})}(g_{n+2}) \leq -T$, then:

$$P[\forall g \in \{g_{n+1},...,g_{n+1}+\omega_n\}, \tau_{(\omega_n+\omega_{n+1},\omega_{n+2})}(g) \geq T] \ll \\ P[\tau_{(\omega_n+\omega_{n+1},\omega_{n+2})}(g_{n+1}) \geq T], \tag{30}$$

where $P[]$ represents the probability.

Although it is possible to think up pathological examples in which $\forall g \in \{g_{n+1},...,g_{n+1}+\omega_n\}, \tau_{(\omega_n+\omega_{n+1},\omega_{n+2})}(g) \geq T$, we have never observed it in any realization of the null-model or the real data for that matter. In contrast, it often happens that $\tau_{(\omega_n+\omega_{n+1},\omega_{n+2})}(g_{n+1}) \geq T$.

Jump discontinuities can become significant after merging. Case 2: bordering a discontinuity that is not significant. Next we introduce a property of the null model similar to Property 4.

Given a segmentation with jump discontinuities $\{\tau_{(\omega_n,\omega_{n+1})}(g_n), \tau_{(\omega_{n+1},\omega_{n+2})}(g_{n+1}), \tau_{(\omega_{n+2},\omega_{n+3})}(g_{n+2})\}$ and an arbitrary positive thresholds T.

- Property 5: If $\tau_{(\omega_n,\omega_{n+1})}(g_n) < T$, $\tau_{(\omega_{n+1},\omega_{n+2})}(g_{n+1}) < T$ and $\tau_{(\omega_{n+2},\omega_{n+3})}(g_{n+2}) < T$, then

$$P[\forall g \in \{g_{n+1}, ..., g_{n+1} + \min(\omega_n + \omega_{n+1}, \omega_{n+2})\}, \tau_{(\omega_n+\omega_{n+1},\omega_{n+2})}(g) \geq T] \\ \ll P[\tau_{(\omega_n+\omega_{n+1},\omega_{n+2})}(g_{n+1}) \geq T], \tag{31}$$

Note that this scenario is quite similar to the one in Property 4. The major difference here is that we don't require $\tau_{(\omega_{n+2},\omega_{n+3})}(g_{n+2})$ to be below the threshold $-T$. Due to the relaxed constraint, we need to extend the domain in which we search for a down-crossing from the width w_n to $\min(\omega_n+\omega_{n+1}, \omega_{n+2})$. The threshold T is large in the sense that segmentation at iteration k only resulted in jump discontinuities lower than T in the neighborhood of g_{n+1}. $\min(\omega_n + \omega_{n+1}, \omega_{n+2})$ is equal to the width of one of the lobes in the kernel corresponding to the scale $w = (\omega_L, \omega_R)$, where $\omega_L = \omega_n + \omega_{n+1}$ and $\omega_R = \omega_{n+2}$. Without loss of generality, let us assume it is w_L. Property 5 is justified by the fact that t_{g_0} and $t_{g_0+w_L}$ is weakly correlated in the null and because T is high in the neighborhood. The weak correlation stems from the fact that the kernel is anti-correlated with itself when shifted by w_L probes:

$$\int_{-\infty}^{\infty} k_w(g_0 - g)k_w(g_0 + w_L - g)dg \quad < \quad 0 \tag{32}$$

As with Property 4, it is possible to think up pathological examples in which $\forall g \in \{g_{n+1}, ..., g_{n+1} + \min(\omega_n + \omega_{n+1}, \omega_{n+2})\}, \tau_{(\omega_n+\omega_{n+1},\omega_{n+2})}(g) \geq T$. However we never observed this in realizations of the null-model.

Invariance in the signs of τ when merging segments. It is easy to prove that:

$$t_{(\omega_{n-1},\omega_n+\omega_{n+1})}(g_n) \quad = \quad t_{(\omega_{n-1},\omega_n)}(g_{n-1}) + \alpha t_{(\omega_n,\omega_{n+1})}(g_n), \tag{33}$$

where $\alpha = \frac{\omega_{n+1}}{\omega_n+\omega_{n+1}} < 1$. From this it follows that:

- Property 6: If $|\tau_{(\omega_n,\omega_{n+1})}(g_n)| < |\tau_{(\omega_{n-1},\omega_n)}(g_{n-1})|$ then either:

$$\begin{aligned} \text{sign}\big(\tau_{(\omega_{n-1},\omega_n+\omega_{n+1})}(g_{n-1})\big) \quad &= \quad \text{sign}\big(\tau_{(\omega_{n-1},\omega_n)}(g_{n-1})\big), \text{ or} \\ |\tau_{(\omega_{n-1},\omega_n+\omega_{n+1})}(g_{n-1})| \quad &\leq \quad |\tau_{(\omega_n,\omega_{n+1})}(g_n)| \end{aligned} \tag{34}$$

What this means is that if we merge two segments that differ with the least significance (remove the discontinuity at g_n), then the new jump discontinuity at g_{n-1}, $\tau_{(\omega_{n-1},\omega_n+\omega_{n+1})}(g_{n-1})$ will have the same sign as the old one $\tau_{(\omega_{n-1},\omega_n)}(g_{n-1})$ or else will be even less significant than the discontinuity that was removed.

Control on the expected number of local maximum segments when clustering. We are now finally in a position to prove that the expected number of local maximum segments will be less than $E/2$ when clustering until all jump discontinuities have a similarity measure below E.

We start with iteration $k = 0$, where every probe is a unique cluster, i.e. the segmented aggregate is exactly the same as the aggregate. At each successive iteration, we merge segments that are separated with the lowest $|\tau| < T$ score. For any threshold T, we can compute $\chi(T)$ at any iteration k and denote it with $\chi^k(T)$. We can also segment on an arbitrary interval of probes $\{q, ..., r\}$ and denote $\chi_{\{q,...r\}}(T)$ at iteration k with $\chi^k_{\{q,...r\}}(T)$

We need to prove that:

$$\forall q \forall r \forall k E[\chi^k_{\{q,\dots,r\}}(T)] \leq (q-r)\Delta\bar{\chi}(T), \tag{35}$$

where $T = -\log(\frac{E}{G-1})$.

For the special case where $q = 0, r = G-1$:

$$\begin{aligned} E[\chi^k_{\{0,\dots,G-1\}}(T)] &\leq (G-1)\Delta\bar{\chi}(T) && \text{Eq. 35} \\ &= (G-1)e^{-T} && \text{Eq. 27} \\ &= (G-1)e^{\log(E/(G-1))} \\ &= E \end{aligned} \tag{36}$$

If we then choose the smallest k for which all τ values at the jump discontinuities are significant, we know that the expected number of local extrema is less than or equal to E. As we observed, the expected number of local maximum segments will be less than or equal to $E/2$.

To prove Equation 35 we use double induction. First on the number of probes $P = r - q + 1$:

Induction hypothesis 1:

$$r - q + 1 < P \implies \forall k E[\chi^k_{\{q,\dots,r\}}(T)] \leq (q-r)\Delta\bar{\chi}(T) \tag{37}$$

From this we need to prove that it also holds for $r - q + 1 = P$. We do so by induction on k.

Suppose $k = 0$. This is the case where we have not yet started merging segments. Every probe is a unique segment. At this point all τ's are at the same scale $w = (1, 1)$. It follows directly

from the definition of the expected Euler characteristic at a fixed scale that:

$$E[\chi^0_{\{q,...,r\}}(T)] \leq (q-r)\Delta\bar{\chi}(T) \tag{38}$$

Now suppose the statement is true for a fixed k.

Induction hypothesis 2:

$$r - q + 1 = P \implies E[\chi^k_{\{q,...,r\}}(T)] \leq (q-r)\Delta\bar{\chi}(T) \tag{39}$$

We need to prove that it also holds for $k+1$. For this we will consider three unique scenarios in which segments are merged from iteration k to $k+1$. All other possible scenarios that can be conceived are either straight forward or strictly symmetric. Without loss of generality we will assume $q = 0$ and $r = P-1$ (we simply relabel the indices and this is legal due to Property 1 and 2), that the discontinuity locations at iteration k will be at $\{g_0, g_1, ...g_{n-1}, g_n, g_{n+1}, ...\}$ and that it is discontinuity g_n that will be merged in iteration $k+1$.

Scenario 1. This scenario is depicted in Supplementary Fig. 8. This scenario is based on two criterion. First, we assume that the right most significant discontinuity g_L before g_{n-1} ($L = \max\{i < n-1 : |\tau_{(\omega_i,\omega_{i+1})}(g_i)| \geq T\}$) has $\tau_{(\omega_L,\omega_{L+1})}(g_L) \geq T$. Second, we assume $\tau_{(\omega_{n+2},\omega_{n+3})}(g_{n+2}) \leq -T$.

If $|\tau_{(\omega_{n-1},\omega_n)}(g_{n-1})| \geq T$ and $|\tau_{(\omega_{n+1},\omega_{n+2})}(g_{n+1})| \geq T$, then due to Property 6, $\chi^{k+1}_{\{0,...,P-1\}}(T) \leq \chi^k_{\{0,...,P-1\}}(T)$ and by induction hypothesis 2, $E[\chi^{k+1}_{\{0,...,P-1\}}(T)] \leq (P-1)\Delta\bar{\chi}(T)$. Therefore, without loss of generality, let us assume $|\tau_{(\omega_{n+1},\omega_{n+2})}(g_{n+1})| < T$. In this case:

$$\begin{aligned} \chi^{k+1}_{\{0,...,P-1\}}(T) \leq\ & \chi^s_{\{0,...,g_{n-1}\}}(T) + \\ & \chi^t_{\{g_n,...,P-1\}}(T) + \\ & 2U(\tau_{(\omega_n+\omega_{n+1},\omega_{n+2})}(g_{n+1}) \geq T) \end{aligned} \tag{40}$$

Here, s and t are the number of iterations required to segment on the intervals $\{0, ..., g_{n-1}\}$ and $\{g_n, ..., P-1\}$ respectively when considered as separate problems. $U()$ is the indicator function with range $\{0, 1\}$. Due to Property 4, we know that $\tau_{(\omega_n+\omega_{n+1},\omega_{n+2})}(g_{n+1}) \geq T$ implies (with high certainty) that there will be a down-crossing in the interval $\{g_{n+1}, ..., g_{n+1}+\omega_n\}$. As a consequence,

$$\begin{aligned} \chi^{k+1}_{\{0,...,P-1\}}(T) \leq\ & \chi^s_{\{0,...,g_{n-1}\}}(T) + \\ & \chi^t_{\{g_n,...,P-1\}}(T) + \\ & 2\chi^{\downarrow}_{\{g_{n+1},...,g_{n+1}+\omega_n\}}(T), \end{aligned} \tag{41}$$

Finally, we can compute the expectation:

$$\begin{aligned}
E[\chi^{k+1}_{\{0,\dots,P-1\}}(T)] &\leq E[\chi^{s}_{\{0,\dots,g_{n-1}\}}(T)] + \\
&\quad E[\chi^{t}_{\{g_n,\dots,P-1\}}(T)] + \\
&\quad 2E[\chi^{\downarrow}_{\{g_{n+1},\dots,g_{n+1}+\omega_n\}}(T)] \\
&\leq (g_{n-1})\Delta\bar{\chi}(T) + \qquad \text{(Induction hypothesis 1)} \\
&\quad (P-1-g_n)\Delta\bar{\chi}(T) + \qquad \text{(Induction hypothesis 1)} \\
&\quad 2(g_n - g_{n-1})\big(\frac{1}{2}\Delta\bar{\chi}(T)\big) \qquad \text{(Eq. 27)} \\
&= (P-1)\Delta\bar{\chi}(T)
\end{aligned} \tag{42}$$

Scenario 2. This scenario is depicted in Supplementary Fig. 9. This scenario is base on three criterion. First, we assume that the right most significant discontinuity g_L before g_{n-1} ($L = \max\{i < n-1 : |\tau_{(\omega_i,\omega_{i+1})}(g_i)| \geq T\}$) has $\tau_{(\omega_L,\omega_{L+1})}(g_L) \geq T$. Second, we assume that the left most significant discontinuity g_R after g_{n+2} ($R = \min\{i > n+2 : |\tau_{(\omega_i,\omega_{i+1})}(g_i)| \geq T\}$) has $\tau_{(\omega_R,\omega_{R+1})}(g_R) \leq -T$. The final criteria is $|\tau_{(\omega_{n+2},\omega_{n+3})}(g_{n+2})| < T$.

If $|\tau_{(\omega_{n-1},\omega_n)}(g_{n-1})| \geq T$ and $|\tau_{(\omega_{n+1},\omega_{n+2})}(g_{n+1})| \geq T$, then due to Property 6, $\chi^{k+1}_{\{0,\dots,P-1\}}(T) \leq \chi^{k}_{\{0,\dots,P-1\}}(T)$ and by induction hypothesis 2, $E[\chi^{k+1}_{\{0,\dots,P-1\}}(T)] \leq (P-1)\Delta\bar{\chi}(T)$. Therefore, without loss of generality, let us assume $|\tau_{(\omega_{n+1},\omega_{n+2})}(g_{n+1})| < T$. In this case:

$$\begin{aligned}
\chi^{k+1}_{\{0,\dots,P-1\}}(T) &\leq \chi^{s}_{\{0,\dots,g_{n-1}\}}(T) + \\
&\quad \chi^{t}_{\{g_{n+1},\dots,P-1\}}(T) + \\
&\quad 2U(\tau_{(\omega_n+\omega_{n+1},\omega_{n+2})}(g_{n+1}) \geq T)
\end{aligned} \tag{43}$$

Note that the only difference between Equation 40 and 43 is that we consider the interval $\{g_{n+1}, \dots, P-1\}$ instead of $\{g_n, \dots, P-1\}$ in the second term. Due to Property 5, we can derive $E[\chi^{k+1}_{\{0,\dots,P-1\}}(T)] \leq (P-1)\Delta\bar{\chi}(T)$ following the same strategy proposed in scenario 1.

Scenario 3. The final scenario that we will consider is depicted in Supplementary Fig. 10. This is the scenario where $\tau_{(\omega_{n-2},\omega_{n-1})}(g_{n-2}) \leq -T$ and $\tau_{(\omega_{n+2},\omega_{n+3})}(g_{n+2}) \leq -T$.

If either $\tau_{(\omega_{n-1},\omega_n)}(g_{n-1}) \geq T$ or $\tau_{(\omega_{n+1},\omega_{n+2})}(g_{n+1}) \geq T$, then $\chi^{k+1}_{\{0,\dots,P-1\}}(T) \leq \chi^{k}_{\{0,\dots,P-1\}}(T)$ and by induction hypothesis 2, $E[\chi^{k+1}_{\{0,\dots,P-1\}}(T)] \leq (P-1)\Delta\bar{\chi}(T)$. Therefore we will assume that neither are above T. In this case:

$$\begin{aligned}
\chi^{k+1}_{\{0,\dots,P-1\}}(T) &\leq \chi^{s}_{\{0,\dots,g_{n-1}\}}(T) + \\
&\quad \chi^{t}_{\{g_{n+1},\dots,P-1\}}(T) + \\
&\quad 2U(\tau_{(\omega_{n-1},\omega_n+\omega_{n+1})}(g_{n-1}) \geq T) + \\
&\quad 2U(\tau_{(\omega_n+\omega_{n+1},\omega_{n+2})}(g_{n+1}) \geq T)
\end{aligned} \tag{44}$$

Due to Property 4, we have:

$$\begin{aligned}\chi^{k+1}_{\{0,\ldots,P-1\}}(T) &\leq \chi^{s}_{\{0,\ldots,g_{n-1}\}}(T) + \\ &\quad \chi^{t}_{\{g_{n+1},\ldots,P-1\}}(T) + \\ &\quad 2\chi^{\downarrow}_{\{g_{n+1},\ldots,g_{n+1}+\omega_n\}}(T) + \\ &\quad 2\chi^{\uparrow}_{\{g_{n-1}-\omega_{n+1},\ldots,g_{n-1}\}}(T) \end{aligned} \quad (45)$$

And the result follows when taking expectations.

Supplementary References

1. Altiok, S. *et al.* Heregulin induces phosphorylation of brca1 through phosphatidylinositol 3-kinase/akt in breast cancer cells. *Journal of Biological Chemistry* **274**, 32274–32278 (1999).

2. Bellacosa, A. *et al.* Molecular alterations of the akt2 oncogene in ovarian and breast carcinomas. *International journal of cancer* **64**, 280–285 (1995).

3. Santarius, T., Shipley, J., Brewer, D., Stratton, M. R. & Cooper, C. S. A census of amplified and overexpressed human cancer genes. *Nature Reviews Cancer* **10**, 59–64 (2010).

4. Teixeira, C., Reed, J. C. & Pratt, M. C. Estrogen promotes chemotherapeutic drug resistance by a mechanism involving bcl-2 proto-oncogene expression in human breast cancer cells. *Cancer research* **55**, 3902–3907 (1995).

5. Sanchez-Garcia, F. *et al.* Integration of genomic data enables selective discovery of breast cancer drivers. *Cell* **159**, 1461–1475 (2014).

6. Forbes, S. A. *et al.* Cosmic: mining complete cancer genomes in the catalogue of somatic mutations in cancer. *Nucleic acids research* **39(Database issue)**, D945 – D950 (2010).

7. Zell, J. A., Tsang, W. Y., Taylor, T. H., Mehta, R. S. & Anton-Culver, H. Prognostic impact of human epidermal growth factor-like receptor 2 and hormone receptor status in inflammatory breast cancer (ibc): analysis of 2,014 ibc patient cases from the california cancer registry. *Breast Cancer Res* **11**, R9 (2009).

8. Holst, F. *et al.* Estrogen receptor alpha (esr1) gene amplification is frequent in breast cancer. *Nature genetics* **39**, 655–660 (2007).

9. Li, Z. *et al.* Etv6-ntrk3 fusion oncogene initiates breast cancer from committed mammary progenitors via activation of ap1 complex. *Cancer cell* **12**, 542–558 (2007).

10. Roidl, A. *et al.* The fgfr4 y367c mutant is a dominant oncogene in mda-mb453 breast cancer cells. *Oncogene* **29**, 1543–1552 (2010).

11. Usary, J. *et al.* Mutation of gata3 in human breast tumors. *Oncogene* **23**, 7669–7678 (2004).

12. Barbacid, M. Ras genes. *Annual review of biochemistry* **56**, 779–827 (1987).

13. Danovi, D. *et al.* Amplification of mdmx (or mdm4) directly contributes to tumor formation by inhibiting p53 tumor suppressor activity. *Molecular and cellular biology* **24**, 5835–5843 (2004).

14. Kauraniemi, P. *et al.* Myb oncogene amplification in hereditary brca1 breast cancer. *Cancer research* **60**, 5323–5328 (2000).

15. Campbell, I. G. *et al.* Mutation of the pik3ca gene in ovarian and breast cancer. *Cancer research* **64**, 7678–7681 (2004).

16. Janknecht, R. On the road to immortality: htert upregulation in cancer cells. *FEBS letters* **564**, 9–13 (2004).

17. Holland, D. G. *et al.* Znf703 is a common luminal b breast cancer oncogene that differentially regulates luminal and basal progenitors in human mammary epithelium. *EMBO molecular medicine* **3**, 167–180 (2011).

18. Jensen, D. E. & Rauscher, F. J. Bap1, a candidate tumor suppressor protein that interacts with brca1. *Annals of the New York Academy of Sciences* **886**, 191–194 (1999).

19. Friedenson, B. The brca1/2 pathway prevents hematologic cancers in addition to breast and ovarian cancers. *BMC cancer* **7**, 1 (2007).

20. Cox, A. *et al.* A common coding variant in casp8 is associated with breast cancer risk. *Nature genetics* **39**, 352–358 (2007).

21. Semb, H. & Christofori, G. The tumor-suppressor function of e-cadherin. *The American Journal of Human Genetics* **63**, 1588–1593 (1998).

22. Borg, Å. *et al.* High frequency of multiple melanomas and breast and pancreas carcinomas in cdkn2a mutation-positive melanoma families. *Journal of the National Cancer Institute* **92**, 1260–1266 (2000).

23. Gayther, S. A. *et al.* Mutations truncating the ep300 acetylase in human cancers. *Nature genetics* **24**, 300–303 (2000).

24. Su, G. H., Song, J. J., Repasky, E. A., Schutte, M. & Kern, S. E. Mutation rate of map2k4/mkk4 in breast carcinoma. *Human mutation* **19**, 81–81 (2002).

25. Mo, D. *et al.* Low pbrm1 identifies tumor progression and poor prognosis in breast cancer. *International journal of clinical and experimental pathology* **8**, 9307 (2015).

26. Chu, E. C. & Tarnawski, A. S. Pten regulatory functions in tumor suppression and cell biology. *Medical Science Monitor* **10**, RA235–RA241 (2004).

27. Murphree, A. L. & Benedict, W. F. Retinoblastoma: clues to human oncogenesis. *Science* **223**, 1028–1033 (1984).

28. Surget, S., Khoury, M. P. & Bourdon, J.-C. Uncovering the role of p53 splice variants in human malignancy: a clinical perspective. *OncoTargets and therapy* **7**, 57–68 (2013).

29. Wu, H.-T., Hajirasouliha, I. & Raphael, B. J. Detecting independent and recurrent copy number aberrations using interval graphs. *Bioinformatics* **30**, i195–i203 (2014).

30. Schouten, P. *et al.* Robust brca1-like classification of copy number profiles of samples repeated across different datasets and platforms. *Molecular Oncology* 1274–86 (2015).

31. Picard, F. *et al.* Joint segmentation, calling, and normalization of multiple cgh profiles. *Biostatistics* 413–28 (2011).

32. Talevich, E., Shain, A. H., Botton, T. & Bastian, B. C. Cnvkit: Copy number detection and visualization for targeted sequencing using off-target reads (2015). URL `http://biorxiv.org/content/early/2014/11/25/010876`.

33. Santarius, T., Shipley, J., Brewer, D., Stratton, M. & Cooper, C. A census of amplified and overexpressed human cancer genes. *Nature Reviews Cancer* 59–64 (2010).

34. Fungtammasan, A., Walsh, E., Chiaromonte, F., Eckert, K. & KD, M. A genome-wide analysis of common fragile sites: what features determine chromosomal instability in the human genome? *Genome Research* 993–1005 (2012).

35. Schlicker, A., Michaut, M., Rahmann, R. & Wessels, L. Oncoscape: Exploring the cancer aberration landscape by genomic data fusion. *Submitted* (2015).

36. Worsley, K. J., Evans, A. C., Marrett, S. & Neelin, P. A three-dimensional statistical analysis for cbf activation studies in human brain. *J Cereb Blood Flow Metab* **12**, 900–918 (1992). URL `http://dx.doi.org/10.1038/jcbfm.1992.127`.

37. Worsley, K. J. Estimating the number of peaks in a random field using the hadwiger characteristic of excursion sets, with applications to medical images. *The Annals of Statistics* **23**, pp. 640–669 (1995). URL `http://www.jstor.org/stable/2242356`.

38. Worsley, K. J. *et al.* A unified statistical approach for determining significant signals in images of cerebral activation. *Hum Brain Mapp* **4**, 58–73 (1996). URL `http://dx.doi.org/3.0.CO;2-O`.

39. Adler, R. J. & Hasofer, A. M. Level crossings for random fields. *The Annals of Probability* **4**, 1–12 (1976).

40. van Dyk, E., Reinders, M. J. & Wessels, L. F. A scale-space method for detecting recurrent dna copy number changes with analytical false discovery rate control. *Nucleic acids research* **41**, e100–e100 (2013).
