# Supplementary material for: RUBIC identifies driver genes by detecting recurrent DNA copy number breaks
Source: Nat Commun. 2016 Jul 11;7:12159. doi: 10.1038/ncomms12159 (PMC4942583; doi:10.1038/ncomms12159)
Supplement: Peer review file [file ncomms12159-s14.pdf]

Transferred manuscripts:

Editorial Note: this manuscript has been previously reviewed at another journal that is not operating a transparent peer review scheme. This document only contains reviewer comments and rebuttal letters for versions considered at Nature Communications.
